# Loss of XIST lncRNA unlocks stemness and cellular plasticity in ovarian cancer

**DOI:** 10.1073/pnas.2418096121

**Published:** 2024-11-15

**Authors:** Ikrame Naciri, Minzhi Liang, Ying Yang, Heather Karner, Benjamin Lin, Maria De Lourdes Andrade Ludena, Eric A. Hanse, Alfredo Lebron, Olga V. Razorenova, Dequina Nicholas, Mei Kong, Sha Sun

**Affiliations:** ^a^Department of Developmental and Cell Biology, Charlie Dunlop School of Biological Sciences, University of California, Irvine, CA 92697; ^b^Department of Molecular Biology and Biochemistry, Charlie Dunlop School of Biological Sciences, University of California, Irvine, CA 92697

**Keywords:** epigenetics, cellular plasticity, XIST, cancer stem cell, ovarian cancer

## Abstract

Plasticity, a key hallmark of cancer, enables cells to transition into different states, driving tumor heterogeneity. This cellular plasticity is associated with cancer progression, treatment resistance, and relapse. Cancer stem cells (CSCs) play a central role in this process, yet the molecular factors underlying cancer cell stemness remain poorly understood. In this study, we explored the role of XIST (X-inactive specific transcript) long noncoding RNA in ovarian cancer stemness and plasticity through in silico and in vitro analyses. We found that XIST is significantly down-regulated in ovarian tumors, with low XIST expression linked to a higher stemness index and lower overall survival. Knocking down XIST in ovarian cancer cells enhanced stemness, particularly increasing mesenchymal-like CSCs, and under hypoxic conditions, it promoted epithelial-like CSC markers. Our findings suggest that XIST loss leads to CSC enrichment and cellular plasticity in ovarian cancer, pointing to potential therapeutic targets for patients with low XIST expression.

## Significance

We show that X-inactive specific transcript (XIST), a long noncoding RNA that controls X chromosome dosage compensation during embryo development, plays a critical role in regulating ovarian cancer cell stemness and plasticity. XIST expression is significantly reduced in ovarian tumors, correlating to a higher stemness index and poorer patient survival. Knockdown of XIST in ovarian cancer cell lines enhances cancer cell stemness and promotes the transition between cancer stem cell subtypes, particularly under hypoxic conditions. These findings suggest that XIST is essential for maintaining cellular identity in ovarian cancer, highlighting its role beyond embryonic development in influencing cancer outcomes.

As a complex genetic and epigenetic disease, cancer has significant intertumor heterogeneity (between patients) and intratumor heterogeneity (within patients) (1). Intratumor heterogeneity refers to the different cell types within a tumor that exhibit distinct phenotypes. Cancer Stem Cells (CSCs), a subpopulation of cancer cells, also known as tumor-initiating cells, play a crucial role in contributing to tumor cell heterogeneity (2). The characteristics of CSCs are similar to those of stem cells (SCs), including self-renewal, tumor initiation potential, and differentiation into multiple tumor cell types (2, 3). These characteristics allow them to sustain tumor development and propagation. Furthermore, CSCs contribute to cellular plasticity in tumors (4). Among all the hallmarks of cancer, cellular plasticity is an emerging one that has gained traction (5). When cancer cells undergo dedifferentiation and return to a progenitor-like/stem-like state, they can overcome antiproliferative signaling that occurs in differentiated cells and promote proliferation (2, 5).

In ovarian cancer, CSCs were identified 18 y ago by isolating tumor-initiating cells from a patient's ascites (6). Since then, molecular characterization of CSCs has gained increasing attention. Ovarian CSC markers have been identified, including the surface markers CD44 and CD24 (7–10). Additionally, CSCs are heterogeneous, with different subtypes exhibiting various phenotypes and markers within the tumor. In breast cancer, it has been shown that proliferative epithelial-like CSCs (E-CSCs) are CD44^low^/CD24^high^ and express the ALDH1A1/A3 enzyme, while quiescent mesenchymal-like CSCs (M-CSCs) are CD44^high^/CD24^low^ (11, 12). In ovarian cancer, CD44 has also been shown to be expressed in M-CSC (13). The significance of CSCs in ovarian cancer has promoted interest in using them as potential therapeutic targets for overcoming resistance to treatment and cancer metastasis (14). It has been suggested that long noncoding RNAs (lncRNAs), a class of transcripts larger than 500 nucleotides, play an important role in CSC maintenance (15, 16). Understanding the regulatory mechanisms of lncRNAs in cancer cell stemness could provide valuable insights into the development of cancer (17). Some lncRNAs have been proposed as promising cancer biomarkers and therapeutic targets due to their documented molecular functions and high level of expression specificity (18, 19). The XIST (X-inactive specific transcript) lncRNA has been linked to cancer in mice and humans (20–23). XIST is one of the first lncRNAs identified and is known as the master regulator of X chromosome inactivation (XCI) (24). XCI is a developmentally regulated dosage compensation process that balances X-linked gene expression between females and males in mammals. Xist-null female mice are born at very low frequency and die before adulthood (25). However, conditional deletion of Xist in the hematopoietic lineage leads to female-specific marrow fibrosis and leukemia (22), which are associated with enrichment of SCs and increased cell cycle activity. It has been proposed that XIST lncRNA can be used as a biomarker for treatment and prognostic marker in breast and testicular cancer (20, 26). XIST is dysregulated in breast cancer and loss of XIST is often associated with breast tumors with poor prognosis (12, 21). The loss of XIST can trigger X chromosome reactivation, leading to overexpression of X-linked genes, which causes cancer progression (27). However, XIST loss does not result in massive X chromosome reactivation in human mammary SCs; only a few genes are reactivated, including the chromatin mediator MED14. Overexpression of MED14 interferes with differentiation and homeostasis in mammary SCs (21). Moreover, the study found that mice carrying an oncogenic mutation suffer from tumorigenesis when XIST is lost in mammary SCs. Additional examples and evidence are required to better understand how XIST regulates cancer cells and how it affects cancer susceptibility, especially in cancers that predominantly affect women (21, 22, 28, 29).

Here, we set out to investigate the expression of XIST lncRNA in ovarian tumors and elucidate its role in ovarian cancer cellular plasticity. By analyzing the downregulation of XIST associated with poorly differentiated ovarian cancer grades and its correlation with poor overall survival, we identified the significance of XIST in determining cancer cell stemness and cellular characteristics. Our study using XIST knockdown (KD) in ovarian cancer cell lines demonstrated how XIST affects cancer SC heterogeneity and that the loss of XIST lncRNA promotes cancer cell invasion and migration. Ovarian cancer cells with XIST KD also exhibit enhanced resistance to cell death under hypoxia, suggesting that XIST is involved in CSC maintenance and regulation. By controlling the cellular plasticity of ovarian cancer cells, XIST plays a critical role in ovarian cancer progression.

## Results

### XIST Is Down-Regulated in Ovarian Tumors, Which Correlates with a Higher Stemness Index and a Lower Survival Rate.

**Fig. 1.**
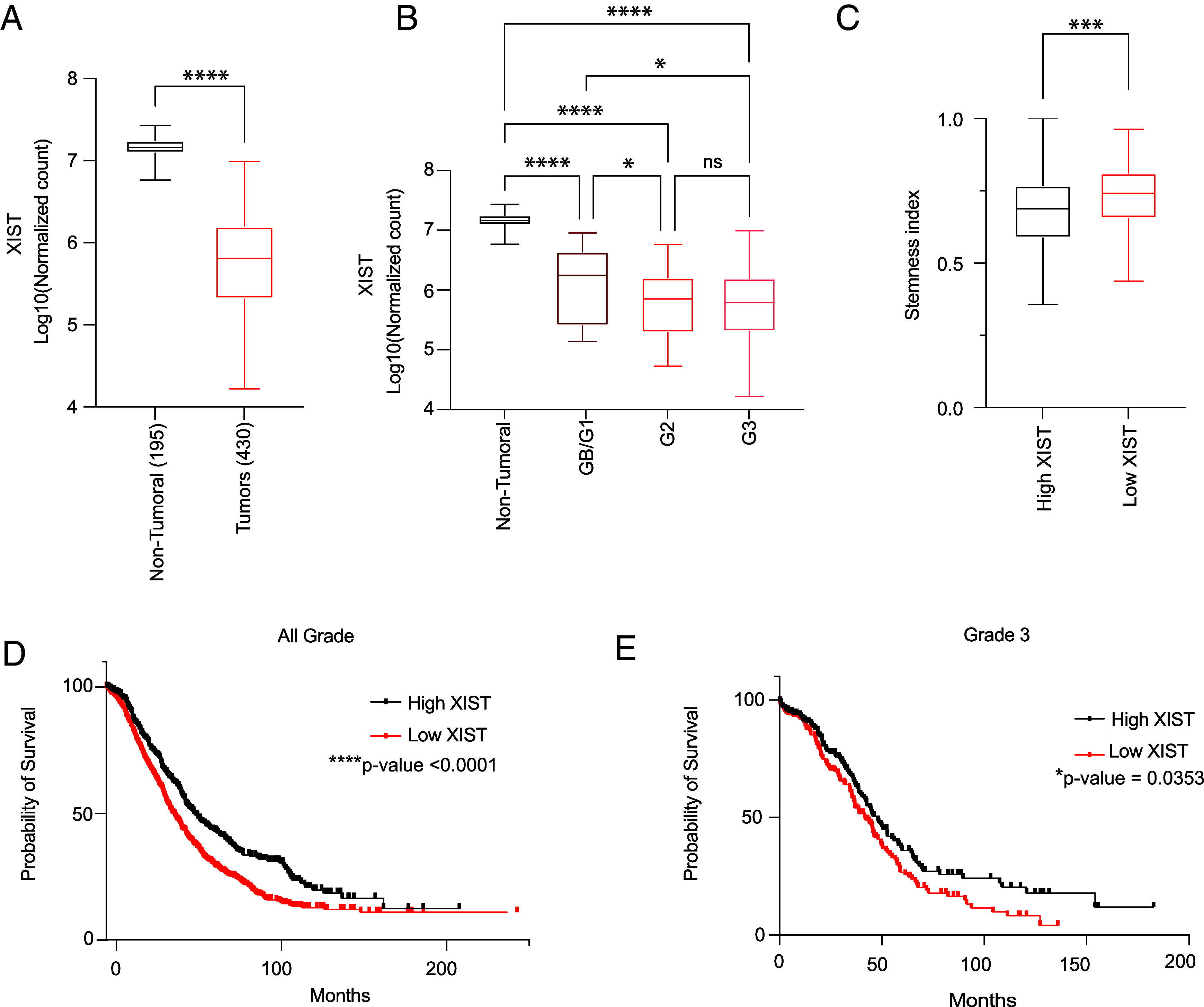
XIST is down-regulated in ovarian tumors in cancer patients, and XIST-low expression is associated with poor prognosis. (*A*) XIST expression in 430 ovarian tumors (TCGA cohort) compared to 195 normal ovaries (GTEx database). (*B*) XIST expression in ovarian tumors sorted by grade compared to normal ovaries (GB: Grade borderline, G2: Grade 2; G3: Grade 3). (*C*) Stemness index of ovarian tumors with high (*Upper* quartile) vs. low XIST (*Lower* quartile) expression. (*D*) Kaplan–Meier curve showing survival of patients with ovarian tumors of low and high XIST expression. (*E*) Kaplan–Meier curve showing survival of patients with Grade 3 ovarian tumors of low and high XIST expression. (*A*–*C*) The statistical analyses were performed with a two-tailed *t* test (* for *P* < 0.05, ** for *P* < 0.01, **** for *P* < 0.0001, ns: non-significant).

To determine XIST expression patterns in ovarian tumors, we used The Cancer Genome Atlas (TCGA) and Genotype-Tissue Expression (GTEx) database (30, 31). Our study compared the expression of XIST in 430 tumor samples and 195 normal ovarian tissues. In contrast to nontumor normal tissue samples, tumor samples had a 10-fold lower expression of XIST (Fig. 1*A*). To investigate further, we examined XIST expression across different tumor histological grades. The histological grade indicates cell differentiation, with Grade 1 for well-differentiated tumor cells, Grade 3 for poorly differentiated cells, and Grade Borderline (GB) for noncancerous ovarian tumors. As compared to GB/G1 grade, higher-grade tumors, particularly Grade 3 (G3), showed significantly lower expression of XIST (*P* < 0.05 for G3 vs. GB/G1 and G2 vs. GB/G1) (Fig. 1*B*).

The lncRNA XIST controls XCI (X chromosome inactivation) in all somatic cells, so we investigated whether XIST downregulation was associated with XCI regulation in ovarian cancer cells. XIST and its flanking regulatory lncRNA genes, such as JPX (Just Proximal of Xist) and FTX (lncRNA Five Prime to Xist), determine the initiation of XCI as well as the decision to silence one of the two X-chromosomes in female cells (32–36). We examined the expression profiles of JPX and FTX, which positively regulate XIST. Both lncRNAs were expressed in ovarian tumors at a twofold lower level compared to nontumoral samples (*SI Appendix*, Fig. S1 *A* and *B*), suggesting that XCI elements were down-regulated in ovarian cancer cells. To determine whether XIST, JPX, and FTX downregulation could be caused by chromosomal deletions, we also examined Copy Number Variation (CNV) across ovarian cancer grades. XIST, JPX, and FTX copy numbers showed no significant differences by grade (*SI Appendix*, Fig. S1 *C*–*E*), suggesting that gene regulatory changes and transcriptional repression were more likely causes of reduced XIST expression in ovarian tumors than chromosomal deletion.

As Grade 3 ovarian tumors showed low XIST expression and previous reports have shown that XIST regulates breast cancer SCs (12, 21), we investigated whether ovarian tumors with low XIST expression had a more undifferentiated cellular state. Using an mRNA stemness index developed by Malta et al. (37), which utilizes a machine-learning algorithm to extract transcriptomic signatures from various SC databases, we evaluated the stemness index in TCGA ovarian cancer patient samples. We validate the stemness index by examining the mRNA stemness index by grade. Grade 3 tumors displayed the highest stemness index, consistent with the histopathology grading for poorly differentiated cancer cells (*SI Appendix*, Fig. S1*F*). Then, an mRNA stemness index is calculated for each subgroup of tumors belonging to the first quartile (high) and the last quartile (low) of XIST expression. In contrast to samples with high XIST expression, samples with low XIST expression showed a higher mRNA stemness index (Fig. 1*C*), suggesting the presence of more cancer SCs in tumors with low XIST expression.

As Grade 3 tumors are associated with increased aggressiveness and unfavorable prognosis, we investigated whether the expression level of XIST could impact patient outcomes (38) We used the Kaplan–Meier plotter, a web database that combines gene expression data from multiple sources and clinical information to generate Kaplan–Meier curves (39), which showed that patients with low XIST expression had a significantly shorter overall survival time (40.43 vs. 53.27 mo, Fig. 1*D*). Within Grade 3 tumors, lower XIST expression was also associated with poor survival (41.63 vs. 48.20 mo, Fig. 1*E*). Overall, results from our in silico analysis demonstrated a significant downregulation of XIST in ovarian cancer patient samples, particularly in higher-grade tumors. The downregulation of XIST was associated with a higher stemness index and an unfavorable prognosis, suggesting that XIST plays a role in regulating ovarian cancer SCs.

### XIST KD in Ovarian Cancer Cell Lines Induces Cell Morphological Changes, Increases Cell Nucleus Size, and Enhances CSC Population.

**Fig. 2.**
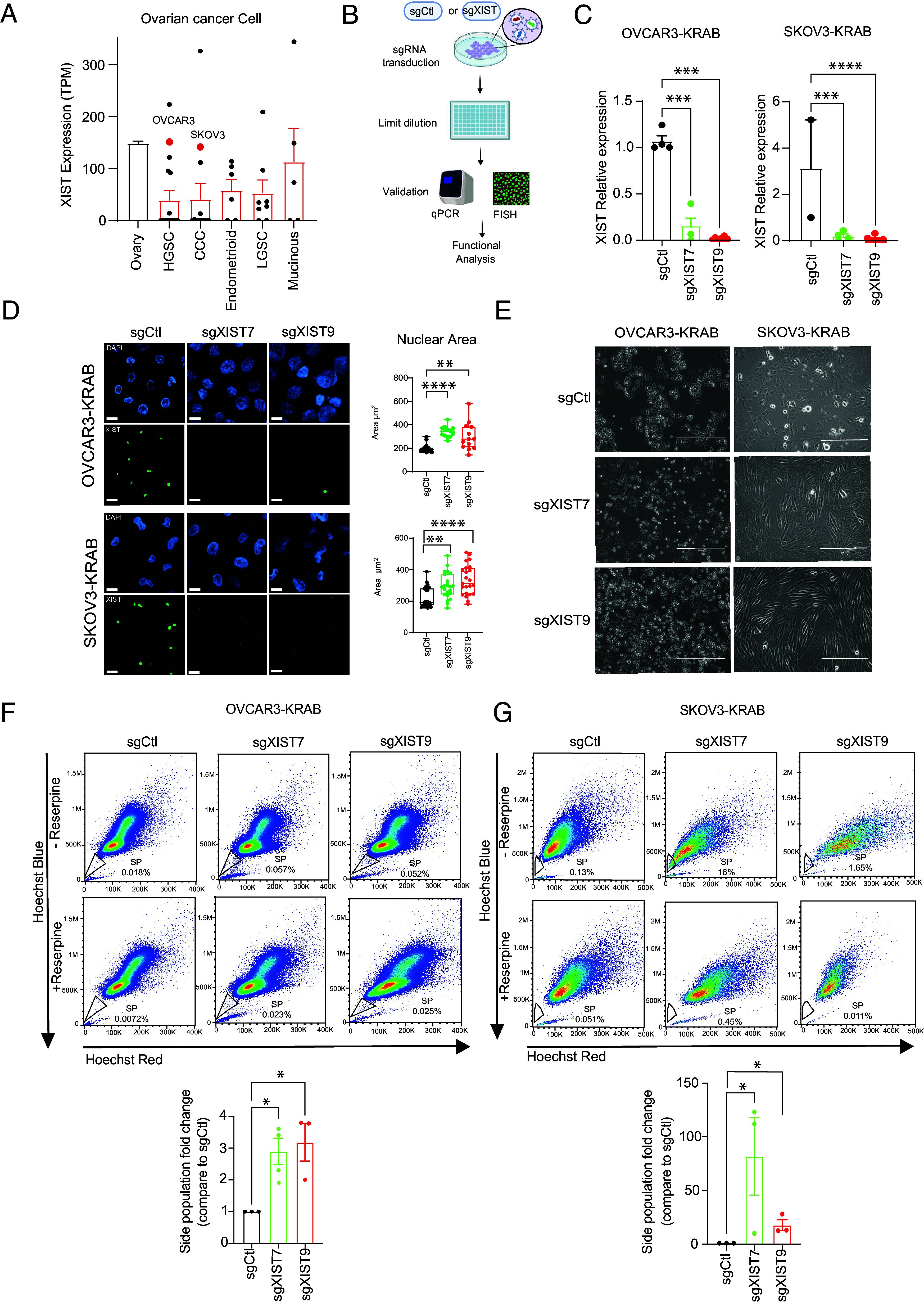
XIST expression in ovarian cancer cell lines and effects of XIST KD. (*A*) XIST expression in ovarian cancer cell lines from CCLE sorted by ovarian cancer subtypes: HGSC, CCC, Endometrioid, LGSC (Low-Grade Serous Carcinoma), and Mucinous. OVCAR3 and SKOV3 show high expression of XIST compared to other ovarian cancer cell lines (red dots). (*B*) Scheme of XIST KD in ovarian cancer cell line using CRISPRi technology. (*C*) XIST expression analysis by RT-qPCR after KD of XIST and clonal selection in OVCAR3-KRAB and SKOV3-KRAB. The statistical analyses were performed with a two-tailed ANOVA test. (*D*) FISH of XIST lncRNA in OVCAR3-KRAB (*Upper*) and SKOV3-KRAB (*Lower*). The histograms on the right represent nucleus area measurement in OVCAR3-KRAB (*Upper*) and SKOV3-KRAB (*Lower*) after XIST KD with sgXIST7 and sgXIST9. Two-tailed one-way ANOVA statistical test. (Scale bar, 10 µm.) (*E*) Brightfield images of OVCAR3-KRAB and SKOV3-KRAB clonal cell lines. (Scale bar, 200 µm.) (*F*) Side population assay in OVCAR3-KRAB after XIST KD in clonal cells. *Upper* panels represent results without ABC transporter inhibitor Reserpine, and *Bottom* panels represent results from cells treated with 15 µM of Reserpine (negative control). Side population was gated by performing the experiment with an ABC transporter inhibitor Reserpine. (*G*) Side population assay in SKOV3-KRAB after XIST KD in clonal cells. *Upper* panels represent results without ABC transporter inhibitor Reserpine, and *Bottom* panels represent results from cells treated with 15 µM of Reserpine (negative control). Side population was gated by performing the experiment with an ABC transporter inhibitor Reserpine. (*C*–*G*) The statistical analyses were performed with a two-tailed *t* test. (* for *P* < 0.05, ** for *P* < 0.01, **** for *P* < 0.0001, ns: nonsignificant). All experiments have been performed at least 3 times.

To better understand the role of XIST in ovarian cancer cell stemness, we examined XIST expression in ovarian cancer cell lines through the CCLE (Cancer Cell Line Encyclopedia) database (40). There are several biological subtypes of ovarian cancer cells, with High-Grade Serous Carcinoma (HGSC) being the most prevalent subtype, accounting for approximately 70% of ovarian cancer cases. Clear Cell Carcinomas (CCC) account for about 10% of all cases, and they are the most aggressive subtype (41). XIST expression was low across all biological subtypes, as observed in ovarian patient samples. Nevertheless, OVCAR3 and SKOV3, belonging to the HGSC and CCC subtypes, respectively, exhibited XIST expression similar to normal ovary XIST expression (>100 transcripts per million, Fig. 2*A*). Our study has used OVCAR3 and SKOV3 as ovarian cancer cell models because these two cell lines have been extensively studied and characterized (42, 43), and both of them express relatively high levels of XIST compared to other ovarian cancer cell lines, which allows us to investigate the function of XIST through gene KD.

We performed XIST-KD in ovarian cancer cells using CRSPRi (CRISPR interference). We first established OVCAR3 and SKOV3 cell lines that stably express a dead Cas9-KRAB (dCas9-KRAB) fusion by lentiviral transduction followed by antibiotic selection; the cell lines are referred to as OVCAR3-KRAB and SKOV3-KRAB hereafter. These cells did not exhibit any changes in cell morphology or XIST expression as a result of the dCas9-KRAB transgene. We then transduced OVCAR3-KRAB and SKOV3-KRAB cell lines with either a control sgRNA (sgCtl) or a sgRNA targeting XIST promoter (sgXIST). We used two sgXIST RNAs, sgXIST7 and sgXIST9, which target different sequence regions of the XIST promoter. We established monoclonal cell lines and selected clones that lost XIST expression (Fig. 2*B*). For OVCAR3-KRAB, we obtained six sgCtl clones, eleven sgXIST7 clones, and fourteen sgXIST9 clones. Eleven of the sgXIST clones had reduced XIST expression by more than 60%. For SKOV3-KRAB, we obtained one sgCtl clone, thirteen sgXIST7 clones, and twenty sgXIST9 clones. Eighteen of the sgXIST clones had reduced XIST expression by more than 60% (*SI Appendix*, Fig. S2 *A* and *B*). In both ovarian cancer cell lines, CRISPRi was effective in knocking down XIST expression. We validated KD efficiencies using RT-qPCR (Fig. 2*C* and *SI Appendix*, Fig. S2*B*) and RNA fluorescent in situ hybridization (FISH) (Fig. 2*D*). In both OVCAR3-KRAB and SKOV3-KRAB cell lines, we observed changes in cell morphology associated with XIST KD. DAPI staining of these cells revealed changes in cell nucleus size. OVCAR3-KRAB and SKOV3-KRAB cells with XIST KD showed larger cell nuclei (Fig. 2*D*). Such morphological phenotypes are typically associated with mesenchymal cells (44), suggesting the possibility that XIST KD might induce an epithelial-to-mesenchymal transition (EMT) in these ovarian cancer cells.

Furthermore, it was found that OVCAR3-KRAB cells with XIST KD were larger and spindle-like than control cells, while SKOV3-KRAB cells with XIST KD were more elongated than control cells, exhibiting more fibroblast-like morphology (Fig. 2*E*). In addition, XIST KD may increase CSCs in ovarian cancer cells based on our finding of a higher stemness index in ovarian tumors with low XIST expression (Fig. 1*C*). We performed side population assay (45) to determine whether CSCs increased after XIST KD. The assay is based on the proprieties of SCs to efflux dye due to their high expression of ABC transporters. After 90 min of staining with Hoechst 33342, cells with low dye staining are defined as the side population (SP) by flow cytometry. We validated our SP gating by performing the same experiment with an ABC transporter inhibitor (Reserpine) that prevents Hoechst from being effluxed from the cells, after the selection of single live cells (*SI Appendix*, Fig. S2). In both SKOV3-KRAB and OVCAR3-KRAB cell lines, SP was significantly enriched after XIST KDs: OVCAR3-KRAB cells showed a threefold increase in SP for both sgXIST7 and sgXIST9 (Fig. 2*F*); SKOV3-KRAB cells showed an 81-fold increase in SP for sgXIST7 and an 18-fold increase in SP for sgXIST9 (Fig. 2*G*). As expected, cells treated with Reserpine showed a depletion of SP in both cell lines. Overall, our findings suggest that ovarian cancer cells with XIST KD undergo EMT and are enriched with CSCs.

### Transcriptome Analysis Reveals Upregulation of CSC-Specific Pathways Associated with XIST KD.

**Fig. 3.**
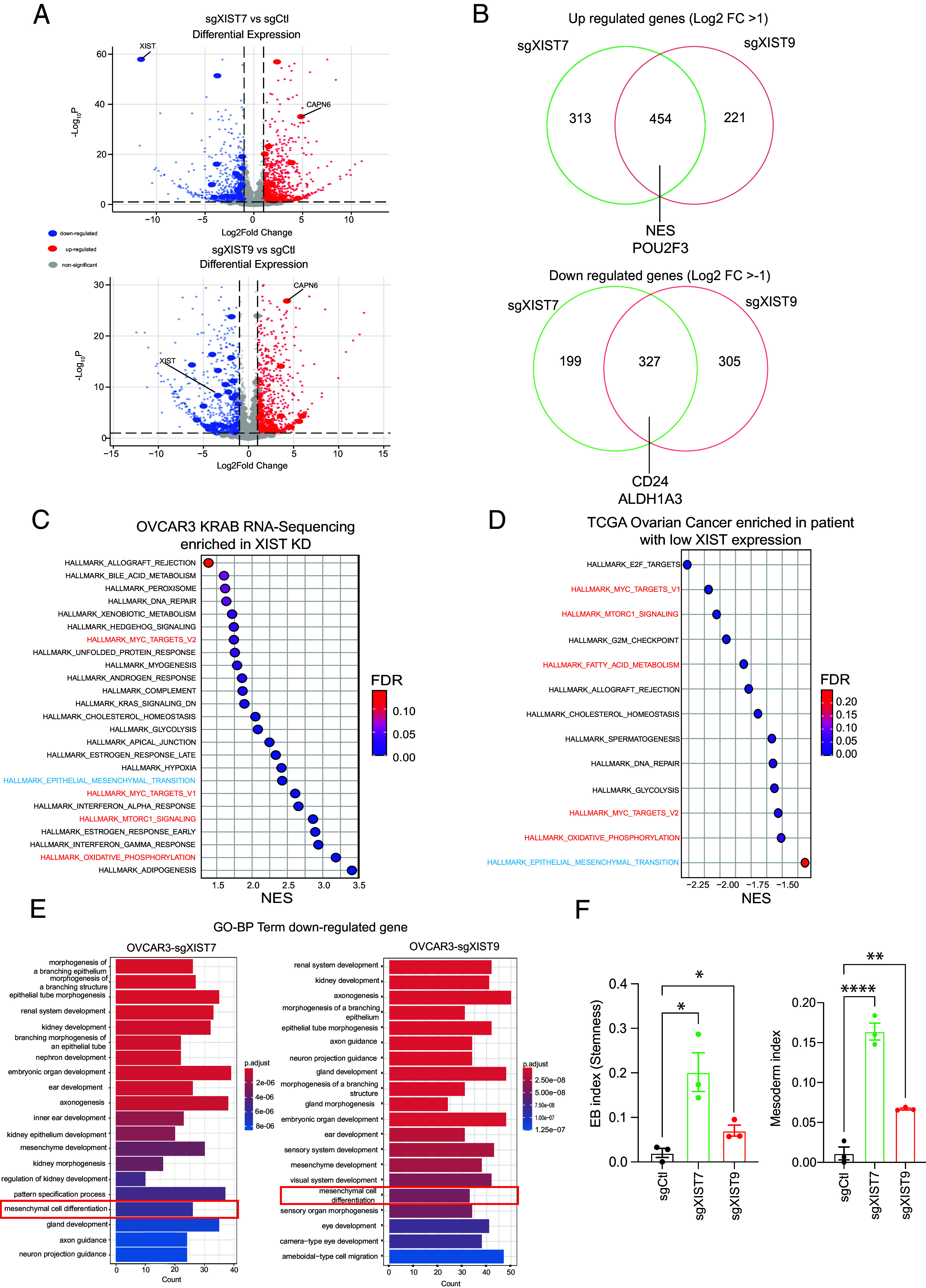
Transcriptome analysis of OVCAR3-KRAB with XIST KD reveals changes in metabolism and cell differentiation. (*A*) Volcano plot showing differential gene expression in OVCAR3-KRAB with sgXIST7 (*Upper*) and sgXIST9 (*Bottom*). Blue dots represent significantly down-regulated genes, red the significantly up-regulated genes (*P*-value cut off: 0.01, log2 fold change cut off: 1), and larger dots represent X chromosome-linked genes. (*B*) Venn Diagram of common significantly up-regulated genes (*Upper*) and down-regulated genes (*Bottom*) in OVCAR3-KRAB with XIST KD (common genes between sgXIST7 and sgXIST9). (*C*) Bubble map of pathways enriched in OVCAR3-KRAB in XIST KD after GSEA. Hallmark gene sets from Human MSigDB Collections have been use for the analyses. Only pathways with an FDR < 0.25 has been plotted. (*D*) Bubble map of pathways enriched in patient samples with low XIST expression after GSEA. Hallmark gene sets from Human MSigDB Collections have been use for the analyses. Only pathways with an FDR < 0.25 has been plotted. (*E*) Gene Ontology analysis of down-regulated genes in OVCAR3-KRAB with sgXIST7(*Left*) and sgXIST9 (*Right*). (*F*) EB Stemness and Mesoderm index in OVCAR3-KRAB. The statistical analyses were performed with a two-tailed *t* test (* for *P* < 0.05, ** for *P* < 0.01, **** for *P* < 0.0001). Three biological replicates were performed for the RNA sequencing experiment.

As part of our investigation into the molecular mechanisms that lead to the phenotypic changes caused by XIST KD, we analyzed the transcriptomes of OVCAR3-KRAB clonal cells by RNA sequencing (RNA-seq). We validated the loss of XIST expression in cells with sgXIST7 and sgXIST9 (Fig. 3*A*). Since XIST is involved in X chromosome inactivation, we first examined whether XIST loss led to the reactivation of X-linked genes. Only a few genes were reactivated: 15 X-linked genes were significantly up-regulated by sgXIST7 and 16 genes by sgXIST9 among 500 X chromosome genes (adjusted p-value cut off = 0.01, log2 fold change cut off = 1) (*SI Appendix*, Fig. S3), consistent with other reports that indicate XIST loss does not cause chromosomal-wide X-linked gene reactivation (21, 22, 46).

Based on differential gene expression analysis, 454 up-regulated and 327 down-regulated genes were identified in both sgXIST7 and sgXIST9 samples compared to sgCtl samples (adjusted *P*-value cut off = 0.01, log2 fold change cut off = 1) (Fig. 3*B* and Dataset S1). In both sgXIST7 and sgXIST9 samples, there was a significant upregulation of NESTIN, which is SC markers of mesenchymal-like CSCs (M-CSC), a subtype of CSC (47). In contrast, the SC surface markers CD24 and ALDH1A3, specific for epithelial-like CSCs (E-CSC) (11, 48), were significantly down-regulated in both sgXIST samples. The loss of XIST in ovarian cancer cells may have changed CSC subtypes.

The Gene Set Enrichment Analysis (GSEA) of mean XIST KD counts, using the Hallmarks gene set from Msigdb, showed an enrichment of pathways up-regulated in CSCs, such as the MTORC1 signaling pathway (NES 2.86) and the MYC target pathway (NES 2.60) (Fig. 3*C*) (49–51). With the TCGA ovarian cancer database, we also identified the same pathway enrichment in ovarian cancer patients using the GSEA preranked option (Fig. 3*D*). Furthermore, the oxidative phosphorylation pathway (OXPHOS), which is highly used by CSC (2), was enriched in OVCAR3-KRAB cells with XIST KD as well as in TCGA ovarian cancer patient samples (Fig. 3 *C* and *D*). XIST KD also resulted in an enrichment of the epithelial-to-mesenchymal hallmark in ovarian cancer cells (Fig. 3 *C* and *D*), consistent with observed morphological changes (Fig. 2*D*). Moreover, Gene Ontology of biological pathway revealed that mesenchymal differentiation was down-regulated in OVCAR3-KRAB cells with XIST KD (Fig. 3*E*), suggesting that the loss of XIST may have promoted a more progenitor/stem-like state in ovarian cancer cells.

We examined the stemness composition of OVCAR3-KRAB cells with XIST KD using mRNA SC-derived embryoid bodies index (EB-index), which reflects the transcriptomic signature of embryonic bodies made of pluripotent SCs, mesoderm, endoderm, and ectoderm progenitor cells (52). This index has also been developed as previously described for the mRNA stemness index. We found that the EB-index is higher in cells with XIST KD than in sgCtl cells (Fig. 3*F*). Therefore, we investigated whether a particular type of progenitor cells was specifically enriched in the XIST KD cells. We observed a higher mesoderm index in OVCAR3-KRAB cells with XIST KD (Fig. 3*F*), suggesting a higher population of mesoderm progenitor cells. As the mesoderm is the ovaries' developmental origin and the primary source of mesenchymal SCs (53, 54), our analysis thus validated that XIST KD increases the mesenchymal SC population in ovarian cancer cells. As a result, the upregulation of mesenchymal stemness markers, epithelial to mesenchymal pathways, and the increase in mesodermal index suggest that loss of XIST promotes ovarian CSCs specifically of the M-CSC subtype.

### XIST KD Induces Mesenchymal-Like CSC Enrichment in Ovarian Cancer Cells.

**Fig. 4.**
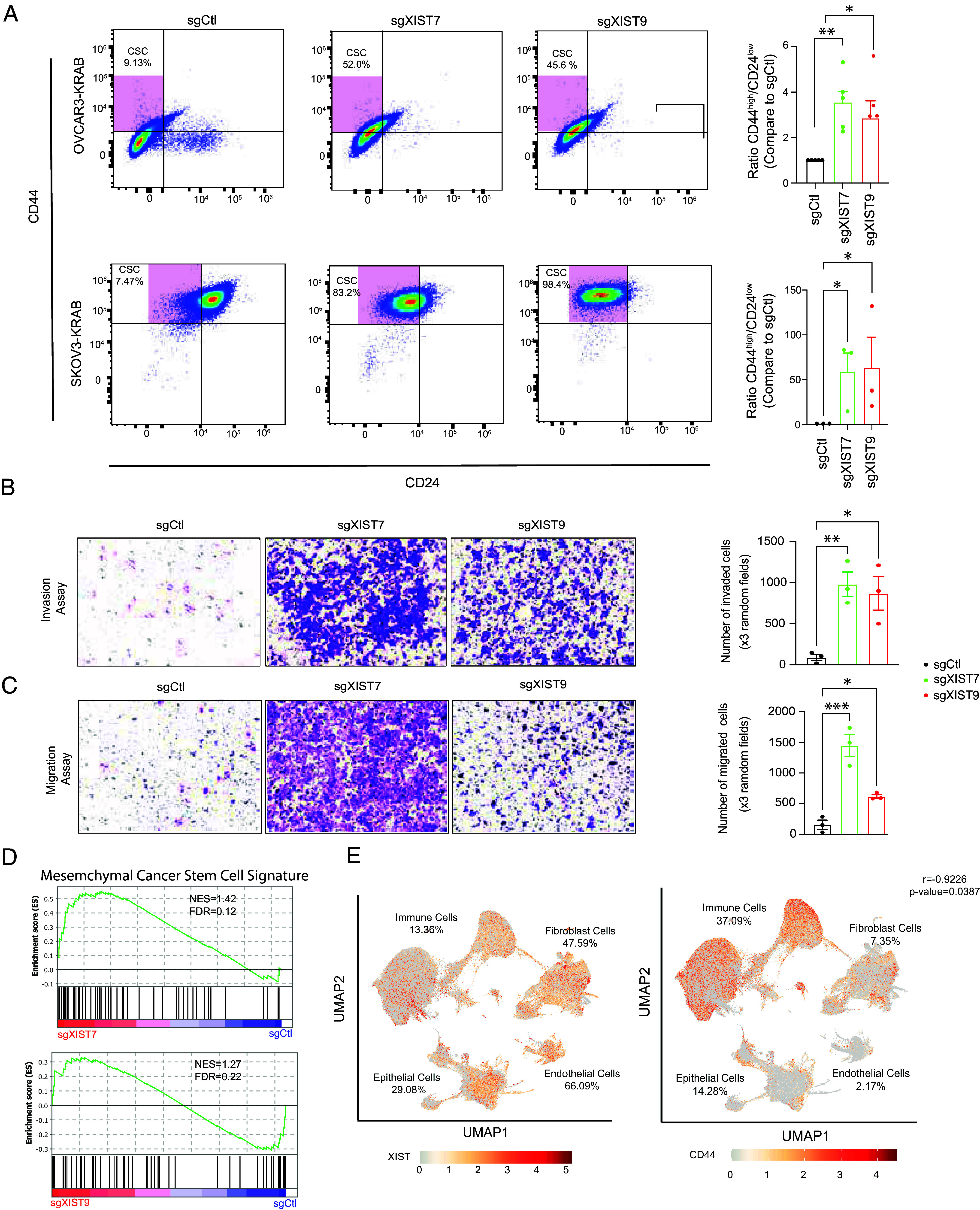
XIST KD leads to M-CSC enrichment in ovarian cancer cell line. (*A*) Flow cytometry analysis of surface markers CD44 and CD24 in OVCAR3-KRAB and SKOV3-KRAB after XIST KD in clonal cells. Statistical analysis two-tailed *t* test. (*B*) OVCAR3-KRAB clonal cell invasion assay. (*C*) OVCAR3-KRAB clonal cell migration assay. The statistical analyses were performed with a two-tailed ANOVA test. (*D*) GSEA plot showing Mesenchymal-like Cancer SC Signature enrichment in OVCAR3-KRAB cell with XIST KD. (*E*) UMAP of XIST (*Left*) and CD44 (*Right*) on ovarian cancer patient single-cell sequencing data. Different cell types as labeled as well as the percentage of cells with gene expression >0. (*A*–*C*) All the experiments have been performed at least three times. (* for *P* < 0.05, ** for *P* < 0.01, **** for *P* < 0.0001).

Mesenchymal-like CSC (M-CSC) subtype has been established in breast cancer: mesenchymal-like CSCs are highly invasive, and express high levels of CD44 cell surface marker but low levels of CD24 marker (CD44^high^/CD24^low^) (11). We used flow cytometry to determine whether ovarian cancer cells with XIST KD exhibited the same mesenchymal-like SC characteristics with CD44^high^/CD24^low^ as breast CSCs. XIST KD resulted in a significant enrichment of the CD44^high^/CD24^low^ population in OVCAR3-KRAB cells (3.21 mean fold increase) and SKOV3-KRAB cells (61.5 mean fold increase), indicating M-CSC enrichment (Fig. 4*A*). Using cell invasion and migration assays, we validated our RNA-seq results which showed higher EMT gene expression (Fig. 3*C*) and determined whether XIST KD cells had invasion and migration characteristics similar to M-CSC. Ovarian cancer SKOV3ip cells are known to be highly invasive (55) and XIST KD did not significantly alter cell invasion or migration in SKOV3-KRAB; however, OVCAR3-KRAB cells with XIST KD exhibited increased invasion and migration (Fig. 4 *B* and *C*). Therefore, loss of XIST in ovarian cancer cells can result in the enrichment of M-CSC with similar characteristics as in breast cancer.

To examine molecular features, we performed GSEA on the OVCAR3-KRAB transcriptome profiles to identify the mesenchymal-like cancer SC signature associated with XIST expression. We took advantage of the metabolic signature of M-CSC defined by Luo et al. and had the signature of mesenchymal-associated cancer cells defined by Fan et al. (11, 56). A list of 55 genes was used to determine whether they were enriched in OVCAR3-KRAB with XIST KD. We found that cells with XIST KD were significantly enriched for M-CSC genes signature for both sgXIST7 (NES = 1.42) and sgXIST9 (NES = 1.27), validating increased mesenchymal-like cancer SC population in ovarian cancer cell with loss of XIST (Fig. 4*D*).

Furthermore, we investigated the potential correlation between XIST and CD44 expression in human ovarian cancer using a single-cell transcriptome database (https://dreamapp.biomed.au.dk/OvaryCancer_DB/) (57), which includes data from over 505,102 single cells from 84 ovarian tumor patients. Our analysis revealed a significant inverse correlation between XIST and CD44 expression across various cell types (R = −0.9226, *P* = 0.0387). Notably, XIST is predominantly expressed in epithelial cancer cells (29.08%), endothelial cells (66.09%), and fibroblasts (47.59%), while CD44 is less expressed in these cell types (14.26%, 2.10%, and 7.36%, respectively) but is highly expressed in immune cells (37.09%) compared to XIST (13.36%). This finding suggests that XIST expression is associated with more differentiated cell states in ovarian cancer and exhibits an anticorrelation with CD44 expression, a pattern consistent with our in vitro observations (Fig. 4*E*).

### XIST KD Enhances Cellular Plasticity in Ovarian Cancer Cells Under Hypoxia.

**Fig. 5.**
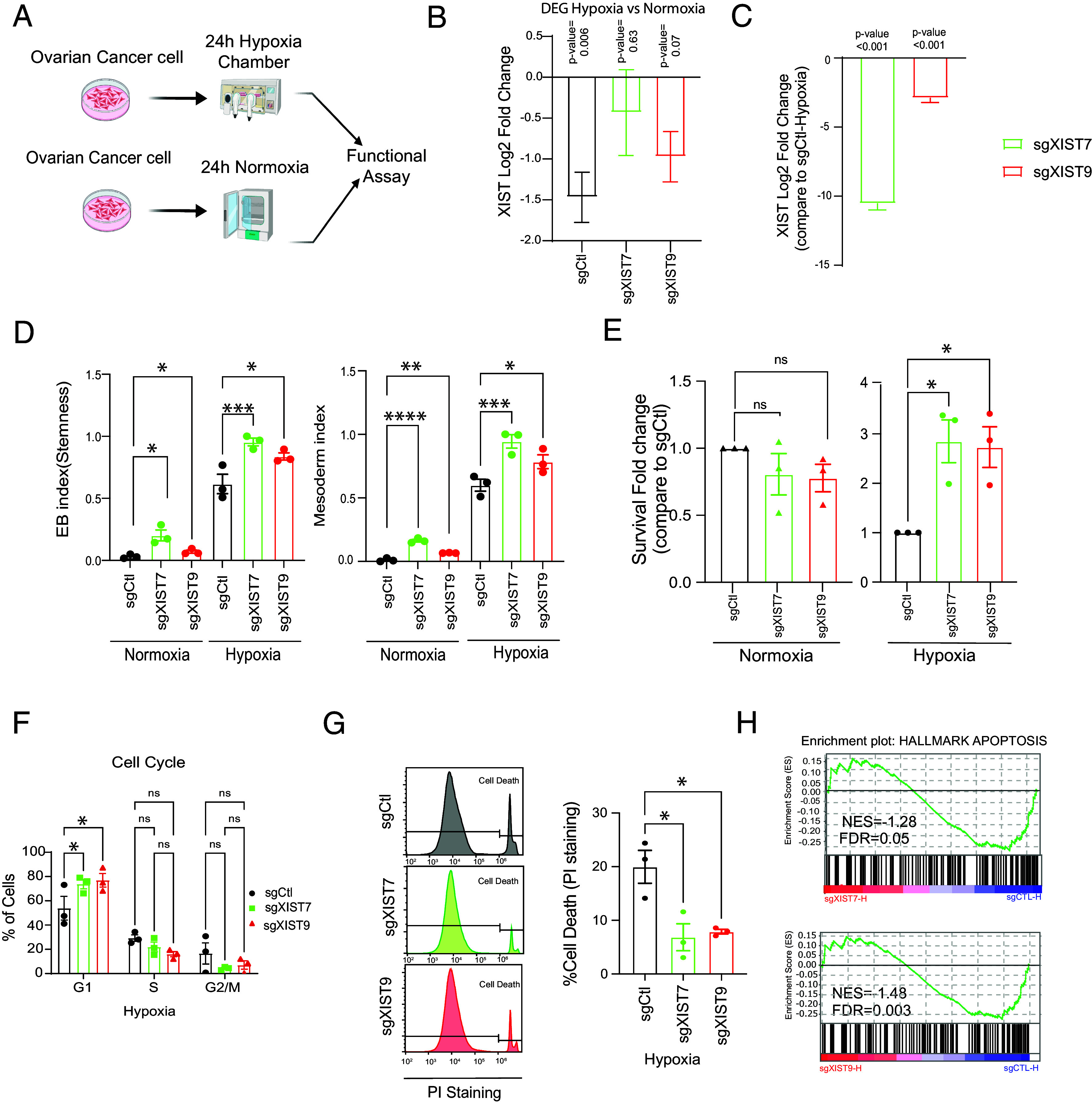
Hypoxia treatment of XIST KD in ovarian cancer cells induces higher stemness and hypoxia-cell death resistance. (*A*) Scheme of the hypoxia experiment strategy. (*B*) Log2 Fold Change of XIST in OVCAR3-KRAB sgCtl-Hypoxia vs. sgCtl-Normoxia, sgXIST7-hypoxia vs. sgXIST7-normoxia, and sgXIST9-hypoxia vs. sgXIST-normoxia after 24 h in hypoxia chamber compared to sgCtl, sgXIST7, and sgXIST9 samples in normoxia respectively assessed by RNA-seq. (*C*) XIST expression in OVCAR3-KRAB with sgXIST7 and sgXIST9 compared to sgCtl after 24 h in hypoxia chamber. (*D*) EB-Stemness and Mesoderm index in OVCAR3-KRAB in normoxia condition and hypoxia condition. (*E*) Survival analysis of OVCAR3-KRAB and SKOV3-KRAB cells with sgCtl or sgXIST after hypoxia treatment assessed by crystal violet assay. (*F*) Flow cytometry analysis of cell-cycle phases (PI staining) of OVCAR3-KRAB cells with XIST KD after hypoxia treatment. (*G*) Flow cytometry analysis of cell death by PI staining after hypoxia treatment in OVCAR3-KRAB cells. (*H*) GSEA of apoptosis pathways in OVCAR3-KRAB cells with sgXIST and sgCtl after hypoxia treatment: *Upper* panel sgXIST7-H (sgXIST7-Hypoxia) vs. sgCtl-H (sgCtl-Hypoxia), *Bottom* panel sgXIST9-H (sgXIST9-Hypoxia) vs. sgCtl-H (sgCtl-Hypoxia). (*D*–*G*) The statistical analyses were performed with a two-tailed ANOVA test (* for *P* < 0.05, ** for *P* < 0.01, **** for *P* < 0.0001). All experiments have been performed at least 3 times.

Tumor hypoxia is a well-known phenomenon in which some parts of the tumors have low oxygen supply due to poor vascularization. Under hypoxia conditions, it has been shown that CSCs are enriched and more resistant to cell death than differentiated cells (58–60). In our study, ovarian cancer cells with XIST KD exhibited enrichment of CSC under normoxia, and we further investigated whether XIST KD would increase CSC enrichment under hypoxia. The OVCAR3-KRAB cells were placed in a hypoxia chamber with 1% oxygen for 24 h and then characterized with a functional assay and transcriptome analysis (Fig. 5*A*).

Based on the RNA-seq data analysis, hypoxia-related genes were enriched in hypoxia compared to normoxia (*SI Appendix*, Fig. S5*A*), confirming the activation of hypoxic pathways. In addition, we found that XIST was down-regulated under hypoxia (Fig. 5*B*). It was not previously known that hypoxia conditions could affect XIST expression, so a reduction in XIST levels in ovarian cancer cells associated with hypoxia suggests that the cellular environment may impact XIST transcriptional regulation and that XIST loss may affect cellular responses to hypoxia. In OVCAR3-KRAB cells with XIST KD, the expression of XIST remained low under hypoxia, as demonstrated by RNA-seq analysis (Fig. 5 *B* and *C*).

To determine whether CSCs are enriched in OVCAR3-KRAB cells under hypoxia, we examined the mRNA SC-derived embryoid body index (EB index: Stemness) (37) in these cells. As shown in Fig. 5*D*, we observed a higher EB stemness index in cell samples under hypoxia than in normoxia, confirming the enrichment of CSC under hypoxia. Moreover, sgXIST samples showed higher EB stemness index values than sgCtl samples under normoxia and hypoxia conditions, confirming that XIST KD enhances the population of CSCs in ovarian cancer cells. We also assessed the mesoderm progenitor enrichment (Mesoderm index) for the developmental germ layer that gives rise to ovaries. As with the EB stemness index, the mesoderm index was higher in cells exposed to hypoxia than normoxia (Fig. 5*D*). In both normoxia and hypoxia conditions, we observed an increase of the mesoderm index in sgXIST samples compared to sgCtl samples, suggesting that XIST KD enhances the mesodermal progenitor-like population in ovarian cancer cells. The results indicate that XIST KD promotes SC and progenitor-like cell populations and may possibly enhance cancer cellular plasticity coping with stressful conditions such as hypoxia.

Then, we investigated whether a higher stemness composition associated with XIST KD led to a better survival rate in OVCAR3-KRAB cells. In normoxia, cell survival rates were not significantly different between cells with sgXIST and cells with sgCtl; however, under hypoxia, OVCAR3-KRAB cells with sgXIST showed significantly higher survival rates (Fig. 5*E*). Analysis of cell cycle and cell death was performed to determine the likely causes of the improved survival. As shown in Fig. 5*F*, sgXIST cells were more prevalent in the G1 phase, suggesting that OVCAR3-KRAB cells with XIST KD remained mostly quiescent under hypoxia. We did not observe any change in cell cycle under normoxia conditions (*SI Appendix*, Fig. S5*B*). In contrast, live cell PI (Propidium Iodide) staining followed by flow cytometry analysis cells revealed less cell death in sgXIST samples than in sgCtl (Fig. 5*G*) under hypoxia. This result is consistent with the RNA-seq data, where GSEA showed an overall downregulation of apoptotic genes indicating less cell death under hypoxia in OVCAR3-KRAB with XIST KD (Fig. 5*H*). Therefore, XIST KD may protect OVCAR3-KRAB cells from hypoxia-induced cell death probably by increasing the CSC population.

### XIST KD Is Involved in CSC Equilibrium.

**Fig. 6.**
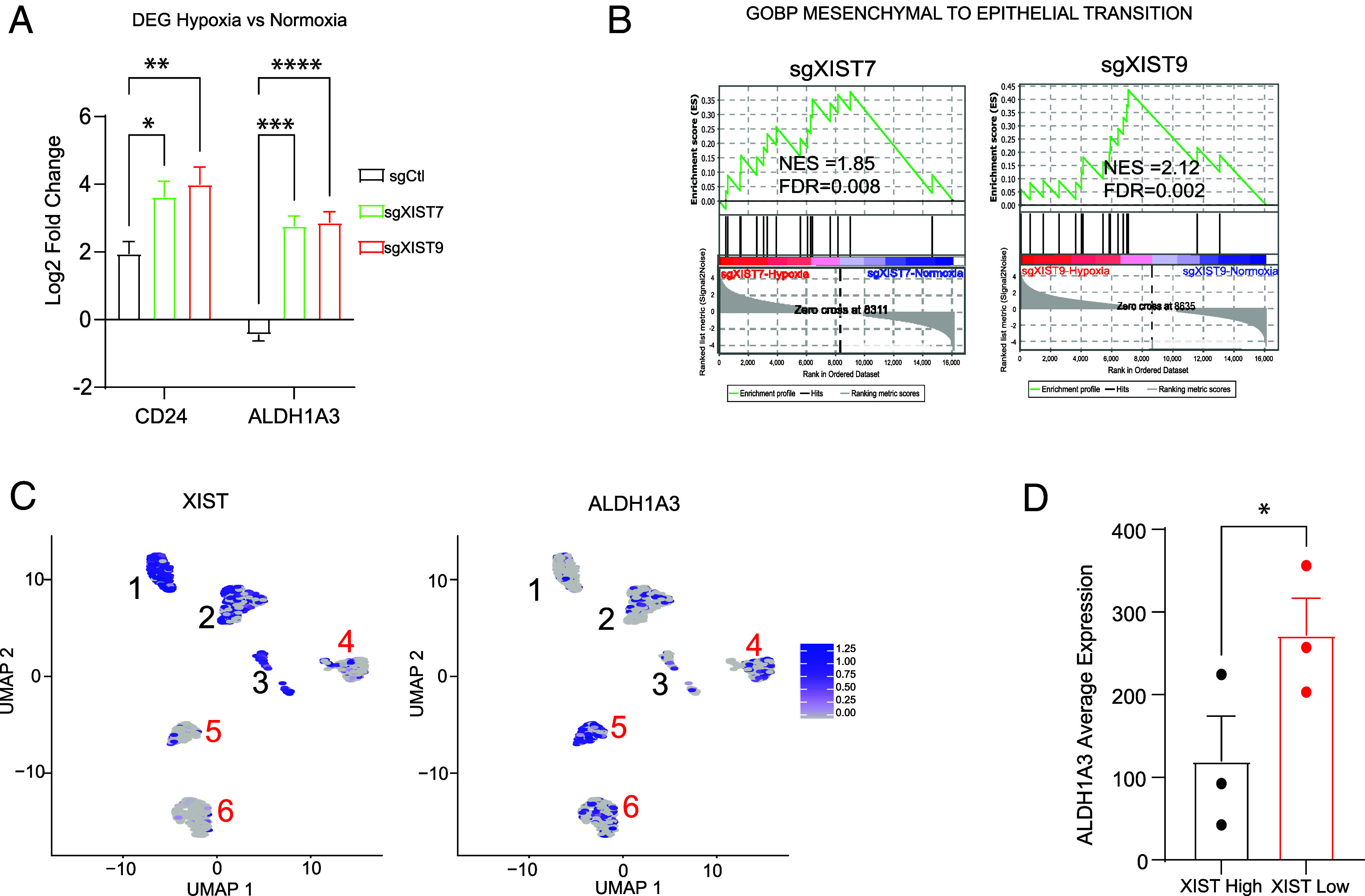
XIST loss enhances cellular plasticity. (*A*) Log2 fold change expression of epithelial markers CD24 and ALDH1A3 after 24 h of hypoxia in OVCAR3-KRAB with sgCtl, sgXIST7, and sgXIST9 vs. sgCtl, sgXIST7, and sgXIST9 respectively in normoxia condition. (*B*) GSEA of OVCAR3-KRAB with sgXIST7-H (sgXIST7-Hypoxia) and sgXIST9-H (sgXIST9-Hypoxia) vs. sgCtl, sgXIST7, and sgXIST9 in normoxia condition. (*C*) UMAP of XIST (*Left*) and ALDH1A3 (*Right*) for CD24+/EPCAM+ tumor cells in ovarian cancer patients. (*D*) Log2 normalization count of ALDH1A3 expression in clusters with high XIST expression and clusters with low XIST expression. The statistical analyses were performed with a *t* test (* for *P* < 0.05, ** for *P* < 0.01, **** for *P* < 0.0001).

Hypoxia impacts CSC equilibrium in breast cancer: CSCs can transdifferentiate from M-CSC to E-CSC (11, 61). We investigated whether a transition from M-CSC to E-CSC occurs in ovarian cancer cells under hypoxia. In ovarian cancer, CD24 has been shown to be expressed in epithelial CSCs (62, 63). Our RNA sequencing data demonstrated an increased expression of CD24 in sgCtl-hypoxia compared to sgCtl-normoxia for OVCAR3-KRAB (Fig. 6*A*). Interestingly, we also observed an increased expression of CD24 in cells with XIST KD after hypoxia treatment, which was twofold greater than the expression increase observed in sgCtl cells (Fig. 6*A*). This confirms the enhancement of E-CSC under hypoxia treatment. We also examined the expression of ALDH1A3, another well-characterized E-CSC marker (11, 48). Similar to CD24, ALDH1A3 expression increased in cells with XIST KD after hypoxia treatment, even though ALDH1A3 was slightly down-regulated in sgCtl OVCAR3-KRAB cells (Fig. 6*A* and Dataset S2). Collectively, these data suggest an increase of epithelial-like CSC presence after hypoxia treatment, with a more significant increase in cells with XIST KD, suggesting a possible transition from M-SCS to E-CSC. We also observed that the specific mesenchymal marker NES was down-regulated in hypoxia compared to normoxia and to the same extent for sgCtl, sgXIST7, and sgXIST9 (*SI Appendix*, Fig. S6*A*), confirming the transition from M-CSC to E-CSC under hypoxia. Moreover, our GSEA revealed an enrichment of the mesenchymal to epithelial transition (MET) pathway in sgXIST7 and sgXIST9 after hypoxia treatment compared to their normoxia condition counterparts (Fig. 6*B*). These data suggest that XIST KD enhances the transition from M-CSC to E-CSC under hypoxia conditions, consistent with higher cellular plasticity after XIST KD.

In addition, we validated our findings using single-cell RNA sequencing data from human patients (64). We analyzed single-cell data from ovarian cancer patients, focusing on cells enriched for the epithelial cancer cell markers CD24+ and EPCAM+ (*SI Appendix*, Fig. S6*B*). Cells were clustered based on their transcriptomic profiles, and the expression levels of XIST and ALDH1A3 were examined (Fig. 6*C*). Three clusters showed high levels of XIST expression (clusters 1, 2, and 3, with log-normalized counts >100), while three other clusters had low XIST expression (clusters 4, 5, and 6, with log-normalized counts <100) (Fig. 6*C*). The expression analysis of ALDH1A3 revealed that clusters with low XIST expression had higher ALDH1A3 expression compared to those with high XIST expression (Fig. 6*D*). These results indicate that epithelial cancer cells with low XIST expression have a higher presence of CSCs, as indicated by the elevated levels of the E-CSC marker ALDH1A3. Altogether, our data suggest that XIST plays a crucial role in cellular plasticity and identity. Downregulation of XIST in ovarian tumors is associated with reduced expression of upstream activators JPX and FTX. Cancer cells that lose XIST expression become less differentiated and more plastic, allowing them to adapt more easily to their environment, which may contribute to cancer progression.

## Discussion

Cell fates can change based on developmental signaling as pluripotent SCs differentiate into a variety of different cell types during development. Cellular plasticity is essential in cancer cells. It is possible for cancer cells to adapt to various environments, metastasize, and resist treatment as they transition between developmental states. CSCs play a vital role in cancer cell plasticity. They are capable of differentiating into various cell types or remaining undifferentiated to survive, proliferate, and migrate. Additionally, CSCs can undergo transdifferentiation, which involves changing from one cell type to another of a different lineage. During the EMT, epithelial cells lose their properties and become mesenchymal, migrate, and invade other organs. The mesenchymal-to-epithelial transition (MET) occurs once the cells invade other organs and transition back to an epithelial state, which allows them to establish new tumor niches and proliferate (65). These processes are facilitated by CSCs, such as M-CSCs and E-CSCs, which maintain cellular plasticity while sustaining tumor growth. It is therefore crucial to understand the development and treatment of cancer by identifying molecular factors that affect CSC and cellular plasticity. In this study, we revealed the function of XIST lncRNA in ovarian cancer cell stemness and plasticity. We demonstrated that XIST was down-regulated in ovarian tumors, resulting in increased cell stemness composition. In two independent ovarian cancer cell lines, knocking down XIST led to enrichment of the M-CSC subtype of CSCs. In a hypoxic environment, however, XIST KD enhanced the E-CSC subtype of CSCs, thus improving survival of these ovarian cancer cells. Our results suggest that XIST plays a critical role in ovarian CSCs and that loss of XIST unlocks cell stemness and plasticity in cancer cells. We also found that XIST downregulation was associated with lower levels of its activators, JPX and FTX, in ovarian tumors. All three genes are located in the same TAD (Topologically Associating Domain) within the XIC (XIST inactivation center) on the X chromosome (66). The reduced expression could be a transcriptional consequence of TAD modulation. However, further studies are needed to determine the regulatory mechanism of XIST expression in ovarian cancer.

Studies of XIST's function in CSC maintenance have been conducted in breast cancer (12, 21). XIST’s role in ovarian cancer has only been explored in the context of therapeutic resistance using transient genetic tools (siRNA, cDNA transfection) (28, 67). Our study here showed how a stable XIST KD in established cell lines affected the outcome of ovarian cancer cells. We observed an enrichment of M-CSCs after XIST KD in ovarian cancer cells. Low levels of XIST in ovarian tumors indicate more M-CSCs, which are more invasive, and are more likely to cause tumors to become aggressive and metastatic, leading to a lower survival rate for patients with low XIST expression. Furthermore, the E-CSC marker ALDH1A3 is down-regulated after XIST KD, suggesting a loss of the E-CSC phenotype. However, our data contrast with findings in breast cancer cell lines, where doxycycline (DOX)-induced XIST KD increases E-CSCs (12). Our study uses a stably established XIST KD cell line, which could explain these differences, indicating that the function of XIST may vary between immediate XIST KD and established XIST KD. Additionally, the effect appears dependent on the cancer subtype, as luminal breast cancer shows no change in mesenchymal-like CSC (M-CSC) proportion (11). XIST could regulate SC-specific pathways through multiple mechanisms. Based on our hypothesis, some X-linked genes that function in SC-specific pathways could be reactivated after XIST KD. Also, XIST can activate or silence genes involved in pluripotency and differentiation by inducing epigenetic modifications, such as chromatin remodeling and DNA methylation; it remains to be elucidated how this directly affects genes on the X chromosome and autosomal genes, which have recently been identified as influenced by XIST in both humans and mice (68, 69).

Our study showed that ovarian cancer cells with XIST KD were more plastic and adapted to hypoxic conditions more easily. XIST KD cells cultured in a hypoxia chamber resisted hypoxia-induced cell death and exhibited upregulation of the E-CSC marker ALDH1A3 compared to the same XIST KD cells in normoxia. Therefore, XIST plays a critical role in modulating CSC cellular states and equilibrium. In particular, we showed that ovarian cancer cells with XIST KD were more likely to transdifferentiate into mesenchymal or epithelial CSC subtypes depending on their environment. The loss of XIST therefore allows cells to transition more easily to different cellular states, enabling them to adapt to various environments and sustain growth. As a result of hypoxia in OVCAR3-KRAB, XIST was down-regulated in sgCtl cells without CRISPRi targeting the XIST promoter. The effect of XIST on cellular plasticity can be explained by the fact that cells down-regulated XIST as a response to hypoxic stress and improved their ability to adapt to the change of environment. As cellular plasticity allows tumor cells to adapt and transdifferentiate in response to various signals and stresses present in a heterogeneous environment, the loss of XIST may result in increased tumor heterogeneity as cancer cells are more adaptable to diverse cell types and characteristics.

Different hypotheses are emerging to understand cancer cell plasticity and tumor heterogeneity; one hypothesis suggests that cancer cells undergo developmental processes that result in a variety of cellular states (4, 70). Cancer cells can advance to a more differentiated state or dedifferentiate into progenitor-like cells within the tumor's organ origin lineage. The expression of XIST in embryonic SCs is low at the early stages of development, but increases as the embryonic cells differentiate into somatic cells (24). To reprogram somatic cells into induced pluripotent SCs (iPSCs), loss of XIST is required during the inverse order of development and cell fate transitions (71). It is consistent with the developmental role of XIST depletion during reprogramming and iPSC process that XIST loss in cancer cells leads to an enrichment of CSCs. While XIST downregulation is associated with cancer treatment resistance (28), it remains unclear whether XIST downregulation occurs early or late in cancer development, or even after cancer treatment. We demonstrate that XIST loss unlocks cancer cell stemness and cellular plasticity in ovarian cancer. Furthermore, the findings may open up therapeutic avenues for patients with low XIST expression of ovarian tumors. The effect of XIST on cellular plasticity may extend beyond breast and ovarian cancer to other cancer types. There is continuing interest in further defining the role of XIST lncRNA in cancer initiation, progression, and therapeutic resistance, especially with the rise of nucleic acid therapeutics such as Antisense Oligonucleotide, RNA interference based therapies, and more recently, mRNA vaccines (72–74).

## Materials and Methods

Full details on the materials and methods are described in *SI Appendix*. Cell lines were cultured in DMEM supplemented with 10% FBS and 1% penicillin/streptomycin. XIST KD was performed using CRISPRi technology. Gene expression analysis was performed by RT-qPCR and RNA sequencing. Side population assay, surface marker analysis, cell cycle analysis, and cell death were assessed by flow cytometry. Invasion and migration assays were performed using the Boyden chamber technique.

## Supplementary Material

Appendix 01 (PDF)

Dataset S01 (XLSX)

Dataset S02 (XLSX)

## Data Availability

RNA-seq data have been deposited at GEO under the accession number GSE271117 (75) and are publicly available as of the date of publication.

## References

[1] F. D. S. E. Melo, L. Vermeulen, E. Fessler, J. P. Medema, Cancer heterogeneity—A multifaceted view. EMBO Rep. **14**, 686–695 (2013). 23846313 10.1038/embor.2013.92 PMC3736134

[2] J.-J. Loh, S. Ma, Hallmarks of cancer stemness. Cell Stem Cell **31**, 617–639 (2024). 38701757 10.1016/j.stem.2024.04.004

[3] E. Batlle, H. Clevers, Cancer stem cells revisited. Nat. Med. **23**, 1124–1134 (2017). 28985214 10.1038/nm.4409

[4] A. S. Patel, I. Yanai, A developmental constraint model of cancer cell states and tumor heterogeneity. Cell **187**, 2907–2918 (2024). 38848676 10.1016/j.cell.2024.04.032 PMC11256907

[5] D. Hanahan, Hallmarks of cancer: New dimensions. Cancer Discov. **12**, 31–46 (2022). 35022204 10.1158/2159-8290.CD-21-1059

[6] S. A. Bapat, A. M. Mali, C. B. Koppikar, N. K. Kurrey, Stem and progenitor-like cells contribute to the aggressive behavior of human epithelial ovarian cancer. Cancer Res. **65**, 3025–3029 (2005). 15833827 10.1158/0008-5472.CAN-04-3931

[7] A. Bartakova , CD44 as a cancer stem cell marker and its prognostic value in patients with ovarian carcinoma. J. Obstetrics Gynaecol. **38**, 110–114 (2018). 10.1080/01443615.2017.1336753 28816557

[8] U. H. M. Sihombing , CD44+/CD24- Expression as predictors of ovarian cancer chemoresistance: Immunohistochemistry and flow cytometry study. J. Egypt. Natl. Canc. Inst. **34**, 44 (2022). 36274112 10.1186/s43046-022-00143-2 PMC13314307

[9] E. Meng , CD44+/CD24− ovarian cancer cells demonstrate cancer stem cell properties and correlate to survival. Clin. Exp. Metastasis **29**, 939–948 (2012). 22610780 10.1007/s10585-012-9482-4

[10] S. Ghafouri-Fard, S. Dashti, M. Farsi, M. Taheri, S. A. Mousavinejad, X-inactive-specific transcript: Review of its functions in the carcinogenesis. Front. Cell Dev. Biol. **9**, 690522 (2021). 34179019 10.3389/fcell.2021.690522 PMC8226258

[11] M. Luo , Targeting breast cancer stem cell state equilibrium through modulation of redox signaling. Cell Metab. **28**, 69–86.e6 (2018). 29972798 10.1016/j.cmet.2018.06.006 PMC6037414

[12] Y. Ma , LncRNA XIST regulates breast cancer stem cells by activating proinflammatory IL-6/STAT3 signaling. Oncogene **42**, 1419–1437 (2023). 36922677 10.1038/s41388-023-02652-3 PMC10154203

[13] R. Strauss , Analysis of epithelial and mesenchymal markers in ovarian cancer reveals phenotypic heterogeneity and plasticity. PLoS One **6**, e16186 (2011). 21264259 10.1371/journal.pone.0016186 PMC3021543

[14] S. Muñoz-Galván, A. Carnero, Targeting cancer stem cells to overcome therapy resistance in ovarian cancer. Cells **9**, 1402 (2020). 32512891 10.3390/cells9061402 PMC7349391

[15] J. S. Mattick , Long non-coding RNAs: Definitions, functions, challenges and recommendations. Nat. Rev. Mol. Cell Biol. **24**, 430–447 (2023), 10.1038/s41580-022-00566-8. 36596869 PMC10213152

[16] Y. Wu, T. Wang, L. Xia, M. Zhang, LncRNA WDFY3-AS2 promotes cisplatin resistance and the cancer stem cell in ovarian cancer by regulating hsa-miR-139-5p/SDC4 axis. Cancer Cell Int. **21**, 284 (2021). 34051810 10.1186/s12935-021-01993-x PMC8164817

[17] M. Huarte, The emerging role of lncRNAs in cancer. Nat. Med. **21**, 1253–1261 (2015). 26540387 10.1038/nm.3981

[18] M. Taheri, A. Safarzadeh, B. M. Hussen, S. Ghafouri-Fard, A. Baniahmad, LncRNA/miRNA/mRNA network introduces novel biomarkers in prostate cancer. Cells **11**, 3776 (2022). 36497036 10.3390/cells11233776 PMC9736264

[19] C. Badowski, B. He, L. X. Garmire, Blood-derived lncRNAs as biomarkers for cancer diagnosis: The good, the bad and the beauty. NPJ Precis. Oncol. **6**, 40 (2022). 35729321 10.1038/s41698-022-00283-7 PMC9213432

[20] F. Xing , Loss of XIST in breast cancer activates MSN-c-met and reprograms microglia via exosomal miRNA to promote brain metastasis. Cancer Res. **78**, 4316–4330 (2018). 30026327 10.1158/0008-5472.CAN-18-1102 PMC6072593

[21] L. Richart , XIST loss impairs mammary stem cell differentiation and increases tumorigenicity through Mediator hyperactivation. Cell **185**, 2164–2183.e25 (2022). 35597241 10.1016/j.cell.2022.04.034

[22] E. Yildirim , Xist RNA is a potent suppressor of hematologic cancer in mice. Cell **152**, 727–742 (2013). 23415223 10.1016/j.cell.2013.01.034 PMC3875356

[23] A. Sadagopan , Somatic XIST activation and features of X chromosome inactivation in male human cancers. Cell Syst. **13**, 932–944.e5 (2022). 36356577 10.1016/j.cels.2022.10.002

[24] B. Payer, J. T. Lee, X chromosome dosage compensation: How mammals keep the balance. Ann. Rev. Genet. **42**, 733–772 (2008). 18729722 10.1146/annurev.genet.42.110807.091711

[25] L. Yang, J. E. Kirby, H. Sunwoo, J. T. Lee, Female mice lacking Xist RNA show partial dosage compensation and survive to term. Genes Dev. **30**, 1747–1760 (2016). 27542829 10.1101/gad.281162.116 PMC5002979

[26] T. Kawakami, K. Okamoto, O. Ogawa, Y. Okada, XIST unmethylated DNA fragments in male-derived plasma as a tumour marker for testicular cancer. Lancet **363**, 40–42 (2004). 14723995 10.1016/S0140-6736(03)15170-7

[27] R. Chaligné , The inactive X chromosome is epigenetically unstable and transcriptionally labile in breast cancer. Genome Res. **25**, 488–503 (2015). 25653311 10.1101/gr.185926.114 PMC4381521

[28] R. Huang, L. Zhu, Y. Zhang, XIST lost induces ovarian cancer stem cells to acquire taxol resistance via a KMT2C-dependent way. Cancer Cell Int. **20**, 436 (2020). 32943985 10.1186/s12935-020-01500-8 PMC7487955

[29] I. Naciri, M. D. Andrade-Ludena, Y. Yang, M. Kong, S. Sun, An emerging link between lncRNAs and cancer sex dimorphism. Hum. Genet. **143**, 831–842 (2023), 10.1007/s00439-023-02620-7. 38095719 PMC11176266

[30] The Cancer Genome Atlas Research Network, Integrated genomic analyses of ovarian carcinoma. Nature **474**, 609–615 (2011). 21720365 10.1038/nature10166 PMC3163504

[31] GTEx Consortium, The genotype-tissue expression (GTEx) project. Nat. Genet. **45**, 580–585 (2013). 23715323 10.1038/ng.2653 PMC4010069

[32] H. Karner , Functional conservation of LncRNA JPX despite sequence and structural divergence. J. Mol. Biol. **432**, 283–300 (2020). 31518612 10.1016/j.jmb.2019.09.002

[33] G. Furlan , The Ftx noncoding locus controls X chromosome inactivation independently of its RNA products. Mol. Cell **70**, 462–472.e8 (2018). 29706539 10.1016/j.molcel.2018.03.024

[34] O. Rosspopoff , Species-specific regulation of *XIST* by the *JPX/FTX* orthologs. Nucleic Acids Res. **51**, 2177–2194 (2023). 36727460 10.1093/nar/gkad029 PMC10018341

[35] S. Sun , Jpx RNA activates Xist by evicting CTCF. Cell **153**, 1537–1551 (2013). 23791181 10.1016/j.cell.2013.05.028 PMC3777401

[36] J.-F. Ouimette, C. Rougeulle, "How many non-coding RNAs does it take to compensate male/female genetic imbalance?" in Non-Coding RNA and the Reproductive System, D. Wilhelm, P. Bernard, Eds. (Advances in Experimental Medicine and Biology, Springer Netherlands, 2016), pp. 33–49. 10.1007/978-94-017-7417-8_3 26659486

[37] T. M. Malta , Machine learning identifies stemness features associated with oncogenic dedifferentiation. Cell **173**, 338–354.e15 (2018). 29625051 10.1016/j.cell.2018.03.034 PMC5902191

[38] L. A. Torre , Ovarian cancer statistics, 2018. CA: A Cancer J. Clin. **68**, 284–296 (2018). 10.3322/caac.21456 PMC6621554 29809280

[39] B. Győrffy, Discovery and ranking of the most robust prognostic biomarkers in serous ovarian cancer. Geroscience **45**, 1889–1898 (2023). 36856946 10.1007/s11357-023-00742-4 PMC10400493

[40] J. Barretina , The cancer cell line encyclopedia enables predictive modelling of anticancer drug sensitivity. Nature **483**, 603–607 (2012). 22460905 10.1038/nature11003 PMC3320027

[41] S. Vaughan , Rethinking ovarian cancer: Recommendations for improving outcomes. Nat. Rev. Cancer **11**, 719–725 (2011). 21941283 10.1038/nrc3144 PMC3380637

[42] C. M. Beaufort , Ovarian cancer cell line panel (OCCP): Clinical importance of in vitro morphological subtypes. PLoS One **9**, e103988 (2014). 25230021 10.1371/journal.pone.0103988 PMC4167545

[43] A. Hallas-Potts, J. C. Dawson, C. S. Herrington, Ovarian cancer cell lines derived from non-serous carcinomas migrate and invade more aggressively than those derived from high-grade serous carcinomas. Sci. Rep. **9**, 5515 (2019). 30940866 10.1038/s41598-019-41941-4 PMC6445084

[44] S. Lamouille, R. Derynck, Cell size and invasion in TGF-β–induced epithelial to mesenchymal transition is regulated by activation of the mTOR pathway. J. Cell Biol. **178**, 437–451 (2007). 17646396 10.1083/jcb.200611146 PMC2064840

[45] A. Golebiewska, N. H. C. Brons, R. Bjerkvig, S. P. Niclou, Critical appraisal of the side population assay in stem cell and cancer stem cell research. Cell Stem Cell **8**, 136–147 (2011). 21295271 10.1016/j.stem.2011.01.007

[46] T. Yang, J. Ou, E. Yildirim, Xist exerts gene-specific silencing during XCI maintenance and impacts lineage-specific cell differentiation and proliferation during hematopoiesis. Nat. Commun. **13**, 4464 (2022). 35915095 10.1038/s41467-022-32273-5 PMC9343370

[47] A. R. Nobre , Bone marrow NG2+/Nestin+ mesenchymal stem cells drive DTC dormancy via TGF-β2. Nat. Cancer **2**, 327–339 (2021). 34993493 10.1038/s43018-021-00179-8 PMC8730384

[48] C. Ginestier , ALDH1 is a marker of normal and malignant human mammary stem cells and a predictor of poor clinical outcome. Cell Stem Cell **1**, 555–567 (2007). 18371393 10.1016/j.stem.2007.08.014 PMC2423808

[49] E. M. Galan-Moya , Endothelial secreted factors suppress mitogen deprivation-induced autophagy and apoptosis in glioblastoma stem-like cells. PLoS One **9**, e93505 (2014). 24682219 10.1371/journal.pone.0093505 PMC3969309

[50] J. Deng , Inhibition of PI3K/Akt/mTOR signaling pathway alleviates ovarian cancer chemoresistance through reversing epithelial-mesenchymal transition and decreasing cancer stem cell marker expression. BMC Cancer **19**, 618 (2019). 31234823 10.1186/s12885-019-5824-9 PMC6591840

[51] H. Ma, T. Tian, Z. Cui, Targeting ovarian cancer stem cells: A new way out. Stem Cell Res. Ther. **14**, 28 (2023). 36788591 10.1186/s13287-023-03244-4 PMC9926632

[52] J. Itskovitz-Eldor , Differentiation of human embryonic stem cells into embryoid bodies compromising the three embryonic germ layers. Mol. Med. **6**, 88–95 (2000). 10859025 PMC1949933

[53] B. Nicol, M. A. Estermann, H. H.-C. Yao, N. Mellouk, Becoming female: Ovarian differentiation from an evolutionary perspective. Front. Cell Dev. Biol. **10**, 944776 (2022). 36158204 10.3389/fcell.2022.944776 PMC9490121

[54] M. A. Vodyanik , A mesoderm-derived precursor for mesenchymal stem and endothelial cells. Cell Stem Cell **7**, 718–729 (2010). 21112566 10.1016/j.stem.2010.11.011 PMC3033587

[55] S.-P. Liu, J.-X. Yang, D.-Y. Cao, K. Shen, Identification of differentially expressed long non-coding RNAs in human ovarian cancer cells with different metastatic potentials. Cancer Biol. Med. **10**, 138–141 (2013). 24379988 10.7497/j.issn.2095-3941.2013.03.003 PMC3860336

[56] H. Fan , Epigenetic reprogramming towards mesenchymal-epithelial transition in ovarian cancer-associated mesenchymal stem cells drives metastasis. bioRxiv [Preprint] (2020). 10.1101/2020.02.25.964197 (Accessed 15 August 2024). PMC7747301 33296650

[57] C. Chai , Single-cell transcriptome analysis of epithelial, immune, and stromal signatures and interactions in human ovarian cancer. Commun. Biol. **7**, 131 (2024). 38278958 10.1038/s42003-024-05826-1 PMC10817929

[58] J. M. Heddleston, Z. Li, A. B. Hjelmeland, J. N. Rich, The hypoxic microenvironment maintains glioblastoma stem cells and promotes reprogramming towards a cancer stem cell phenotype. Cell Cycle **8**, 3274–3284 (2009). 19770585 10.4161/cc.8.20.9701 PMC2825672

[59] B. Keith, M. C. Simon, Hypoxia-inducible factors, stem cells, and cancer. Cell **129**, 465–472 (2007). 17482542 10.1016/j.cell.2007.04.019 PMC3150586

[60] M. Ejtehadifar , The effect of hypoxia on mesenchymal stem cell biology. Adv. Pharm. Bull. **5**, 141–149 (2015). 26236651 10.15171/apb.2015.021 PMC4517092

[61] S. J. Conley , Antiangiogenic agents increase breast cancer stem cells via the generation of tumor hypoxia. Proc. Natl. Acad. Sci. U.S.A. **109**, 2784–2789 (2012). 22308314 10.1073/pnas.1018866109 PMC3286974

[62] S. C. Parte, S. K. Batra, S. S. Kakar, Characterization of stem cell and cancer stem cell populations in ovary and ovarian tumors. J. Ovarian. Res. **11**, 69 (2018). 30121075 10.1186/s13048-018-0439-3 PMC6098829

[63] M. F. Shi , Identification of cancer stem cell-like cells from human epithelial ovarian carcinoma cell line. Cell. Mol. Life Sci. **67**, 3915–3925 (2010). 20549538 10.1007/s00018-010-0420-9 PMC11115598

[64] B. Izar , A single-cell landscape of high-grade serous ovarian cancer. Nat. Med. **26**, 1271–1279 (2020). 32572264 10.1038/s41591-020-0926-0 PMC7723336

[65] B. Bakir, A. M. Chiarella, J. R. Pitarresi, A. K. Rustgi, EMT, MET, plasticity, and tumor metastasis. Trends Cell Biol. **30**, 764–776 (2020). 32800658 10.1016/j.tcb.2020.07.003 PMC7647095

[66] E. P. Nora , Spatial partitioning of the regulatory landscape of the X-inactivation centre. Nature **485**, 381–385 (2012). 22495304 10.1038/nature11049 PMC3555144

[67] Q. Meng, N. Wang, G. Duan, Long non-coding RNA XIST regulates ovarian cancer progression via modulating miR-335/BCL2L2 axis. World J. Surg. Oncol. **19**, 165 (2021). 34090463 10.1186/s12957-021-02274-7 PMC8180121

[68] I. Dror , XIST directly regulates X-linked and autosomal genes in naive human pluripotent cells. Cell **187**, 110–129.e31 (2024). 38181737 10.1016/j.cell.2023.11.033 PMC10783549

[69] S. Yao, Y. Jeon, B. Kesner, J. T. Lee, Xist RNA binds select autosomal genes and depends on Repeat B to regulate their expression. bioRxiv [Preprint] (2024). 10.1101/2024.07.23.604772 (Accessed 1 October 2024). PMC12148325 40488744

[70] Y. Wei , Single-cell analysis and functional characterization uncover the stem cell hierarchies and developmental origins of rhabdomyosarcoma. Nat. Cancer **3**, 961–975 (2022). 35982179 10.1038/s43018-022-00414-w PMC10430812

[71] V. Pasque , X chromosome reactivation dynamics reveal stages of reprogramming to pluripotency. Cell **159**, 1681–1697 (2014). 25525883 10.1016/j.cell.2014.11.040 PMC4282187

[72] C. Liu , mRNA-based cancer therapeutics. Nat. Rev. Cancer **23**, 526–543 (2023). 37311817 10.1038/s41568-023-00586-2

[73] M. Coan, S. Haefliger, S. Ounzain, R. Johnson, Targeting and engineering long non-coding RNAs for cancer therapy. Nat. Rev. Genet. **25**, 578–595 (2024). 38424237 10.1038/s41576-024-00693-2

[74] M. Winkle, S. M. El-Daly, M. Fabbri, G. A. Calin, Noncoding RNA therapeutics—Challenges and potential solutions. Nat. Rev. Drug Discov. **20**, 629–651 (2021). 34145432 10.1038/s41573-021-00219-z PMC8212082

[75] I. Naciri , Loss of XIST lncRNA promotes stemness and cellular plasticity in ovarian cancer [RNA-Seq]. Gene Expression Omnibus. https://www.ncbi.nlm.nih.gov/geo/query/acc.cgi?acc=GSE271117. Deposited 27 June 2024. 10.1073/pnas.2418096121 PMC11588085 39546568

