# Supplementary material for: Loss of XIST lncRNA unlocks stemness and cellular plasticity in ovarian cancer
Source: Proc Natl Acad Sci U S A. 2024 Nov 15;121(47):e2418096121. doi: 10.1073/pnas.2418096121 (PMC11588085; doi:10.1073/pnas.2418096121)
Supplement: Supplementary file 1 — Appendix 01 (PDF) [file pnas.2418096121.sapp.pdf]

PNAS

Supporting Information for
Loss of XIST lncRNA unlocks stemness and cellular plasticity in ovarian cancer.

Ikrame Naciri[1*], Minzhi Liang[1,3], Ying Yang[2], Heather Karner[1,4], Benjamin Lin[1,5], Maria De Lourdes Andrade Ludena[1], Eric Hanse[2], Alfredo Lebron[1], Olga V Razorenova[2], Dequina Nicholas[2], Mei Kong[2], Sha Sun[1*]

* Correspondence: Ikrame Naciri, inaciri@uci.edu; Sha Sun, shasun@uci.edu

This PDF file includes:

Supplementary Materials and Methods
Figures S1 to S6

Other supporting materials for this manuscript include the following:

Datasets S1 to S2

Supplementary Materials and Methods

Cell lines

Human ovarian adenocarcinoma cell line SKOV3iP1 was a cell stock maintained in the Razorenova lab, and OVCAR3 was a generous gift from Dr. Andrej Luptak's lab (University of California, Irvine). Cells were cultured in DMEM medium supplemented with 10% FBS and 1% penicillin/Streptomycin. All the cell lines were cultured in a humidified atmosphere at 37°C under 5 % CO_2.

RNA extraction

RNA extraction was performed using Trizol. Briefly, Trizol was added to the cell and homogenized. Then, chloroform was added to allow phase separation. After centrifugation, isopropanol was added to precipitate RNA. After centrifugation, the pellet was washed three times with 75% Ethanol. Then, the pellet was air-dry and resuspended in Ultrapure water.

RNA sequencing (RNA-seq)

RNA sequencing was performed at the UCI Genomics High-Throughput Facility. 500 ng of RNA was used for sequencing. Quality of the RNA was checked by Bioanalyzer. After rRNA depletion, the sequencing library was constructed using the Illumina TruSeq Stranded Total RNA Ribo-Zero Gold kit. RNA was sequenced using Illumina NovaSeq 600, and around 50 million paired-end reads were sequenced per library.

Bioinformatics analysis

Fastq files were trimmed using the Trimmomatic package, and quality was checked using Fastqc package. Reads were aligned using hisat2 package using hg38 human genome as a reference. Counts were generated using feature counts, and Differential expression was performed with the DeSeq2 package. Mean normalized count list was used for pathway analysis with GSEA (Gene Set Enrichment Analysis) software with Hallmark gene sets from Human MSigDB Collections. Gene Ontology analysis was performed by using the ggprofiler R package.

EB and Mesoderm Index Calculation

Embryoid bodies and the mesoderm index are calculated using machine learning models trained on gene expression profiles, as described by Malta et al (1). The predictive model was trained on pluripotent stem cell samples from the PCBC (Progenitor Cell Biology Consortium) dataset. This model identifies key genes and pathways associated with stemness, pluripotency, and differentiation, assigning scores that indicate how closely a sample resembles an embryoid body or mesodermal lineage. The stemness index was validated with the TCGA PanCancer cohort and independent glioma and breast cancer datasets as described (1). The index was normalized to a [0,1] range to facilitate interpretation and comparison across platforms. We used the functions EB_PCBC_stemSig and MESO_PCBC_stemSig from the TCGAbiolinks package to calculate all the scores.

Cell Cycle Analysis

Cell cycle analysis was performed by staining of DNA content with Propidium Iodide. Briefly, $1x10^6$ was fixed with 70% Ethanol overnight. The next day, cells were washed with cold PBS and stained with Propidium Iodide and RNase A. DNA content was analyzed using NovoCyte Flow cytometer.

RT-qPCR

First-strand cDNA was generated from 1 µg total RNA using Superscript III RT (LifeTechnologies) and Oligo DT for reverse transcription. qPCR was performed using iSYBR Green (BioRad) on a BioRad Real-Time PCR System. Expression values were normalized to the housekeeping genes *TBP* and *GAPDH.*

RNA fluorescent in situ hybridization (FISH)

$2x10^5$ OVCAR3-KRAB and SKOV3-KRAB cells were harvested and fixed onto slides using 4% PFA and stored in 70% ethanol. XIST RNA was hybridized using the RNAscope XIST probe Hs-XIST-C2-Homo sapiens XIST (ACD #311231) and TSA Vivid Fluorophore 520 (ACD #323271) following the RNAscope Multiplex Fluorescent V2 assay manufacturer's instructions in a 40°C incubator. Slides were mounted using ProLong Gold antifade mounting medium. Images were acquired using a Zeiss LSM 900 with Airyscan 2. Nuclei were measured using Imaris 3/4D Image Visualization and Analysis Software Spots and Surfaces module.

Lentiviral Production

HEK293T cell has been used for the production of lentiviral particles. Cells are seeded at 5.10^6 in 10 cm dishes the day before transfection. Cells are transfected with packaging vectors psPAX2 (Addgene #12260), pMD2.G (Addgene #12259), and plasmid of interest using lipofectamine 2000 as a transfection reagent. One day after the media is collected, centrifuge for 5 min at 1000 rpm, then supernatant filtered through 0.45 μm filter. Polybrene is added to the supernatant at 8 μg/ml final concentration before cell infection.

CRISPRi

Stably expressing dCas9-KRAB OVCAR3 and SKOV3 were generated by transduction of Lenti-dCas9-KRAB-blast (Addgene #89567). Blasticidin (10μg/ml) was used to select cells with dCas9-KRAB integration for five days. Then cells were transduced with sgRNA backbone plasmid (lentiGuide-Puro Addgene #52963), where sgRNA targeting XIST promoter has been cloned. (sgXIST7 5'GCAGCGCTTTAAGAACTGAA 3'; sgXIST9 5'GCCATATTTCTTACTCTCTCG 3'). A sgRNA targeting Gal4 promoter has been used as a negative control sgCtl (sgCtl 5'GAA CGA CTA GTT AGG CGT GT 3') (2).Cells were selected with Puromycin (2 μg/ml) for two days.

Side Population assay

OVCAR3-KRAB and SKOV3-KRAB cells were plated in 10 cm cell culture dishes 24 hours before the assay. $2x10^6$ cells for OVCAR3-KRAB and $8x10^5$ cells for SKOV3-KRAB were plate per 10 cm dishes. The next day, cells were harvested and resuspended at a concentration of $1x10^6$ cells/mL in DMEM+ 10% FBS with 5 ug/mL Hoechst 33342,

either alone or with ABC efflux pump inhibitor Reserpine at 15 μM (Sigma). Cells were incubated for 90 minutes at 37°C with shaking every 30 minutes. After incubation, cells were centrifuged and resuspended in PBS+FBS 2%, and dead cells were stained with Propidium iodide (PI) at the final concentration of 2 μg/mL before flow cytometry analysis. Analysis was performed by Agilent NovoCyte 3000, Hoechst dye was exiting by the 405 nm laser, Hoechst Blue was measured by the 445/45 filter, and Hoechst Red by the 675/30 filter. For gating SP population, briefly, we select our cell population with the SSC-H and FSC-H channel, then we select single cells with the SSC-H /SSC-A channel. Next, we select live cells with Propidium Iodide staining. Then SP cells were gated based low stained cells.

Surface Marker Flow Cytometry Analysis

$1x10^6$ cells of OVCAR3-KRAB or SKOV3-KRAB were harvested per sample and washed once with PBS before being stained with the Zombie NIR dye (Biolegend 423105) for cell death exclusion for 20 minutes.After centrifugation, cells were stained with surface markers CD44-PE-Cys5 (Biolegend 103010, 1/400 dilution) and CD24-BV421 (Biolegend 31122, 1/100 dilution) for 20 minutes on ice protected from light, followed by one washed with FACS Buffer (0.1% BSA, 50 mM EDTA, 1X PBS). All data were collected on Northern Lights (Cytek Biosciences). Unstained cells were used to define positively stained cells after the identification of single live cells. The gating strategy example has been exposed in Figures S4A and S4B. Briefly, we select our cell population with the SSC-H and FSC-H channel, then we select single cells with the SSC-H /SSC-A channel. Next, we select live cell with Zombie NIR staining (1/16000). Then

CD24+ and CD44+ cells were gated based on the background signal of the unstained control.

Invasion and Migration Assay

$1x10^5$ OVCAR3-KRAB cells were harvested by trypsinization, washed with PBS and resuspended in 200 µl of serum-free DMEM, and gently added to the upper compartment of the 8 µM insert. 600 µL of DMEM supplemented with 10% FBS was added to the well as an attractant. For the invasion assay, 60 µL ECM gel (Sigma Aldrich E1270) was added to the upper part of the chamber and allowed to solidify before the cells were added. Cells were allowed to migrate and invade for 24 hours before being stained with 0.5% Crystal Violet assay and imaged, and cells were counted.

Single Cell Sequencing analysis

We used the publicly available sequencing dataset under the accession number GSE146026(3).Single-cell RNA sequencing (scRNA-seq) analysis was performed using the Seurat package in R. The data was preprocessed by filtering out cells with an abnormally low or high number of detected genes (nFeature_RNA), high counts (nCount_RNA), or high mitochondrial gene expression (percent.mt) to remove potential doublets or dead cells. After quality control, principal component analysis (PCA) was conducted to reduce the dimensionality of the data. Cells were clustered based on their principal component scores to identify distinct cell populations. These clusters were visualized using Uniform Manifold Approximation and Projection (UMAP) plots. Gene

average expression by cluster was calculated using the function *aggregate expression* from the Seurat package.

REFERENCES

1. T. M. Malta, *et al.*, Machine Learning Identifies Stemness Features Associated with Oncogenic Dedifferentiation. *Cell* **173**, 338-354.e15 (2018).

2. L. A. Gilbert, *et al.*, CRISPR-Mediated Modular RNA-Guided Regulation of Transcription in Eukaryotes. *Cell* **154**, 442–451 (2013).

3. B. Izar, *et al.*, A single-cell landscape of high-grade serous ovarian cancer. *Nat Med* **26**, 1271–1279 (2020).

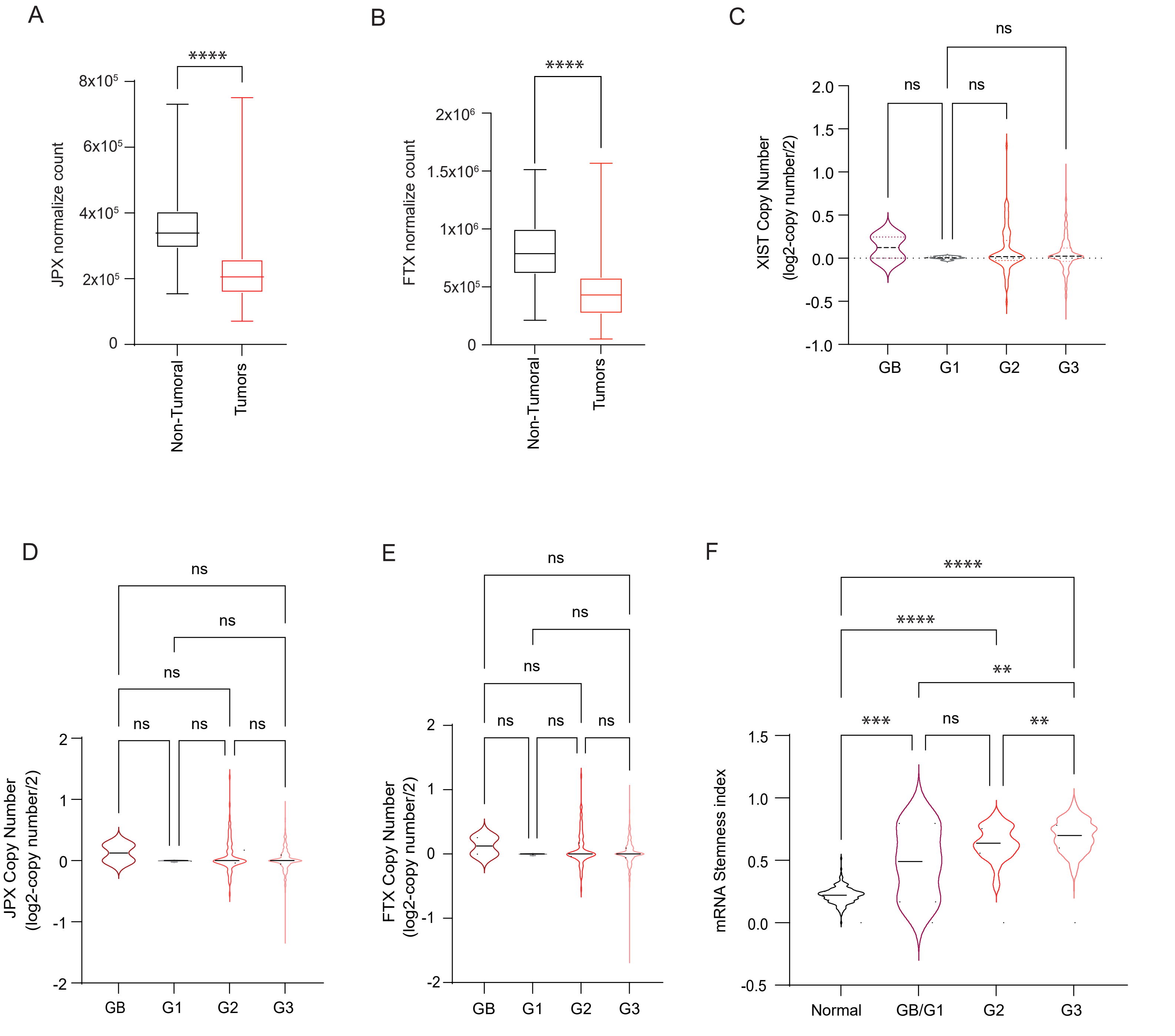

<u>**Supplemental Figure 1:**</u> **(A)** JPX expression in ovarian tumors (430) compared to normal ovaries (195). **(B)** FTX expression in ovarian tumors (430) compared to normal ovaries (195).**(C)** XIST copy number variation in ovarian tumors across grade. **(D)** JPX copy number variation in ovarian tumors across grade. **(E)** FTX copy number variation in ovarian tumors across grade.**(F)** mRNA Stemness index in ovarian tumors across grade. The statistical analyses were performed with a two-tailed t-test (* for P <0.05, ** for P <0.01, **** for P <0.0001, ns: non-significant).

A

Cell line	sgCtl	sgXIST7	sgXIST9
OVCAR3-KRAB	6	11	14
SKOV3-KRAB	1	13	20

B

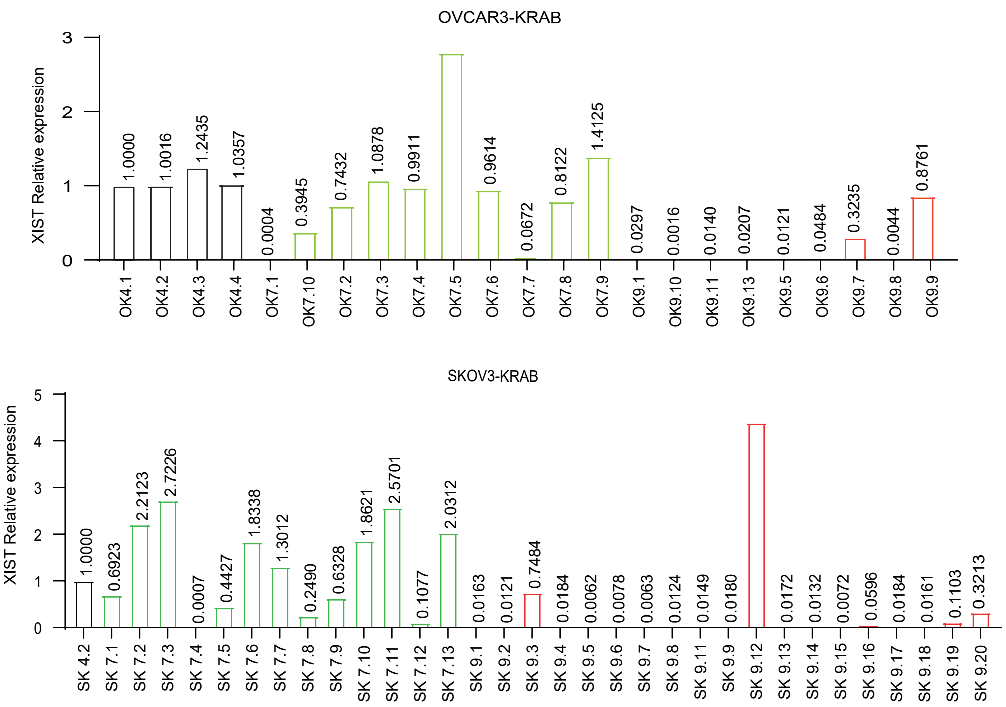

C

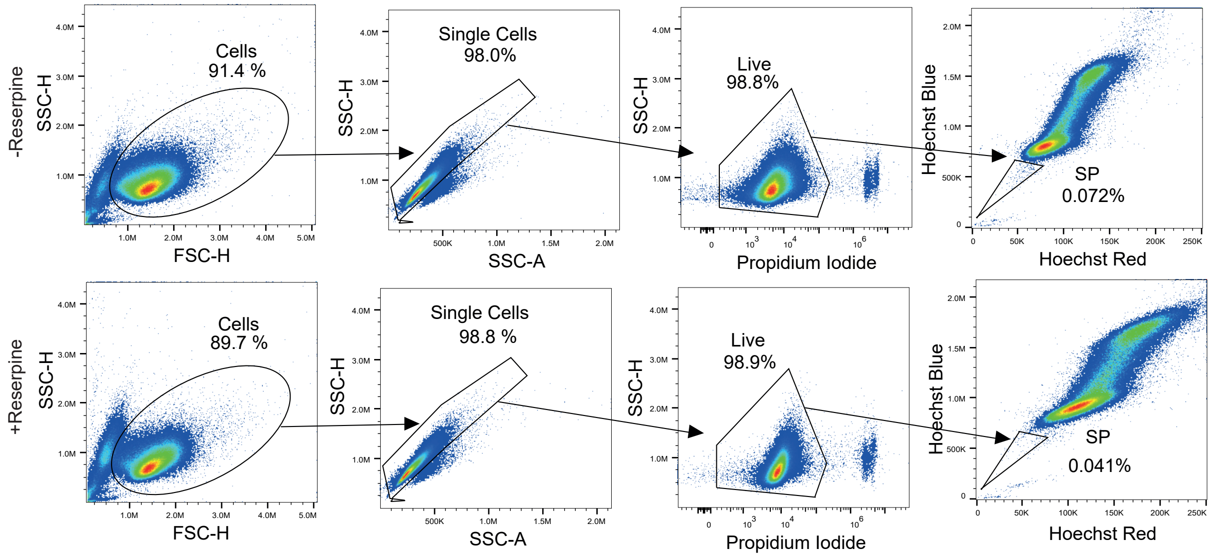

<u>Supplemental Figure 2:</u> (A) Table of clone numbers after limiting dilution in OVCAR3-KRAB and SKOV3-KRAB. **(B)** Relative XIST expression in different OVCAR3-KRAB clones (upper panel) and SKOV3-KRAB clones (bottom panel). **(C)** Example of side population gating strategy.

sgXIST7 vs sgCtl
X linked genes

Gene	log2FoldChange	lfcSE	pvalue	padj
TSC22D3	1.078242	0.281421	0.000127	0.001111
L1CAM	2.835814	0.771926	0.000239	0.001929
ZC3H12B	1.403714	0.415667	0.000733	0.005109
AR	3.894868	0.43449	3.12E-19	2.05E-17
SAT1	1.176848	0.266223	9.85E-06	0.000112
LANCL3	3.023034	0.781172	0.000109	0.00097
CAPN6	4.840833	0.37466	3.44E-38	8.37E-36
TIMP1	1.509528	0.143152	5.36E-26	6.42E-24
REPS2	1.171786	0.309196	0.000151	0.001284
ZDHHC9	1.189897	0.231938	2.89E-07	4.48E-06
ARHGEF6	1.829572	0.291286	3.36E-10	8.18E-09
RPS6KA6	1.122776	0.114117	7.66E-23	7.19E-21
TMEM47	2.377583	0.14504	2.16E-60	1.11E-57
ARMCX3	1.108075	0.342332	0.001209	0.007853
RHOXF1-AS1	4.521983	1.301727	0.000513	0.00376

sgXIST9 vs sgCtl
X linked genes

Genes	log2FoldChange	lfcSE	pvalue	padj
DUSP9	1.03139943	0.164843385	3.93E-10	9.66E-09
L1CAM	2.672680835	0.77834605	0.000595194	0.004319602
AR	3.587737774	0.435041585	1.63E-16	8.21E-15
TENM1	2.222907612	0.552742722	5.78E-05	0.000565656
ZNF711	1.065339781	0.144386426	1.60E-13	6.00E-12
SAT1	1.286660219	0.266393047	1.37E-06	1.91E-05
LANCL3	3.587481048	0.7738364	3.55E-06	4.61E-05
CAPN6	4.26210324	0.376267441	9.61E-30	1.44E-27
ZDHHC9	1.302215228	0.232031865	2.00E-08	3.85E-07
CSAG3	5.606079076	1.384437567	5.14E-05	0.000507689
STS	1.223487474	0.272703197	7.24E-06	8.77E-05
TMEM47	2.86916869	0.144078092	3.08E-88	2.61E-85
RENBP	4.684011764	1.422132694	0.000988945	0.00667568
NHSL2	1.206723741	0.271778498	8.99E-06	0.000106852
CLCN4	1.341415452	0.384078935	0.000478429	0.003583989
RHOXF1-AS1	5.958236654	1.288008922	3.73E-06	4.81E-05

Supplemental Figure 3: Up-regulated X-linked genes after XIST KD in the OVCAR3-KRAB cell line. The genes highlighted in red are specific to each guide.

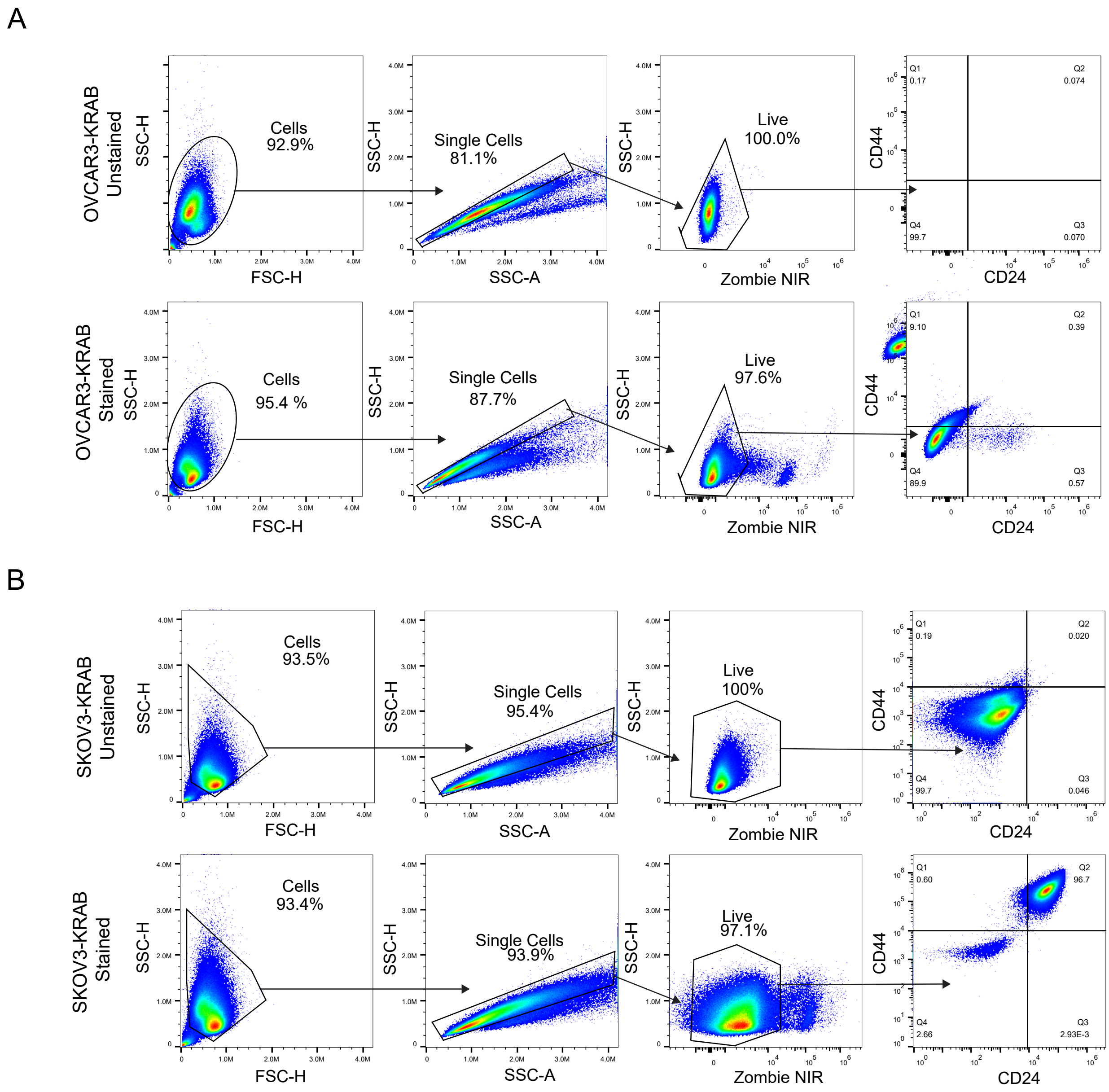

Supplemental Figure 4: **(A)** Example of gating strategy in OVCAR3-KRAB for CD24 and CD44 staining, the top panel represents unstained cells. **(B)** Example of gating strategy in SKOV3-KRAB for CD24 and CD44 staining, the top panel represents unstained cells.

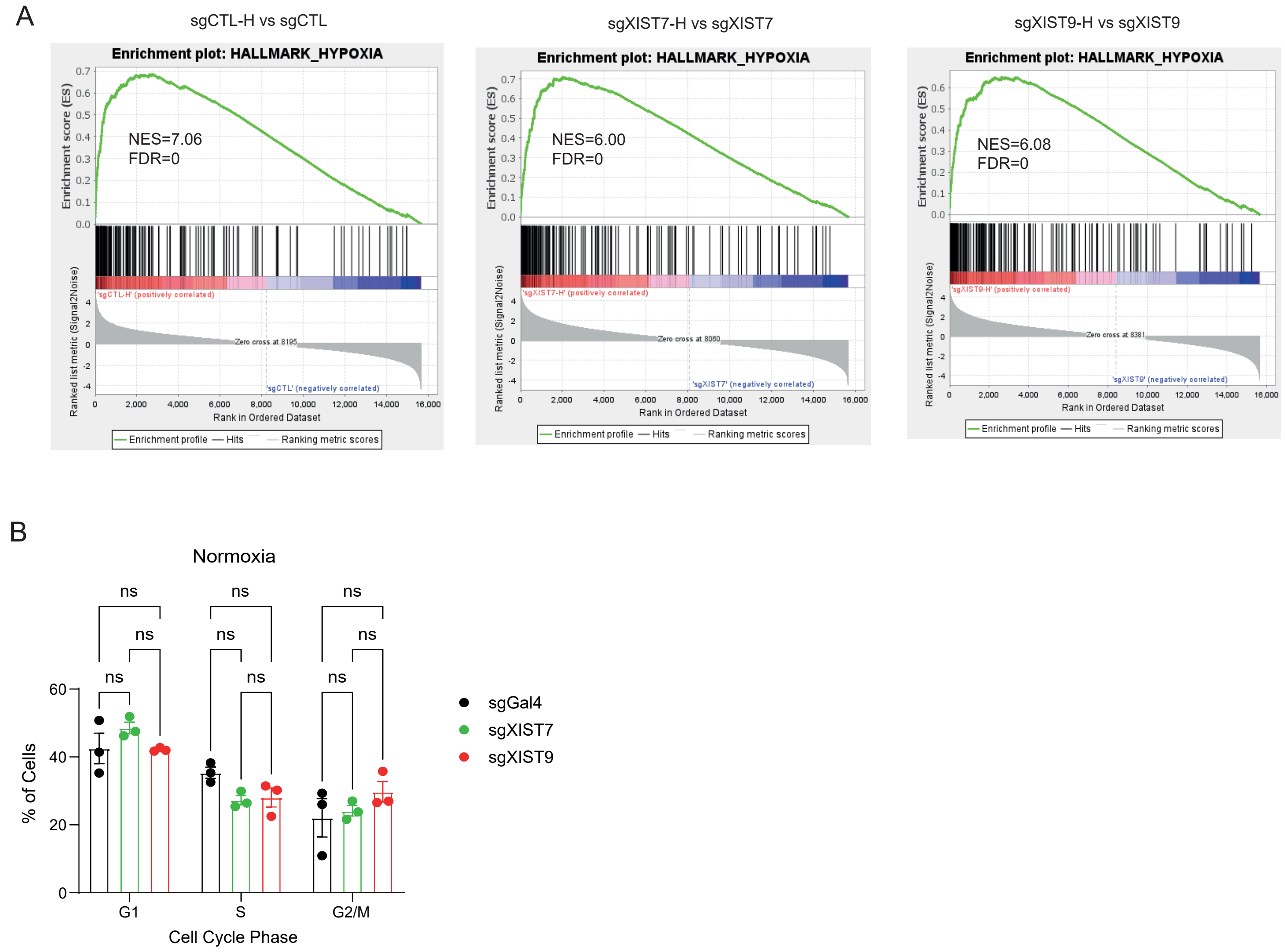

<u>Supplemental Figure 5:</u> (A) GSEA analysis of OVCAR-KRAB transcriptome showing enrichment of hypoxia genes after 24h of hypoxia incubation in sgCtl or sgXIST. **(B)** Flow cytometry analysis of cell-cycle phases (PI staining) of OVCAR3-KRAB with or without XIST KD under normoxia

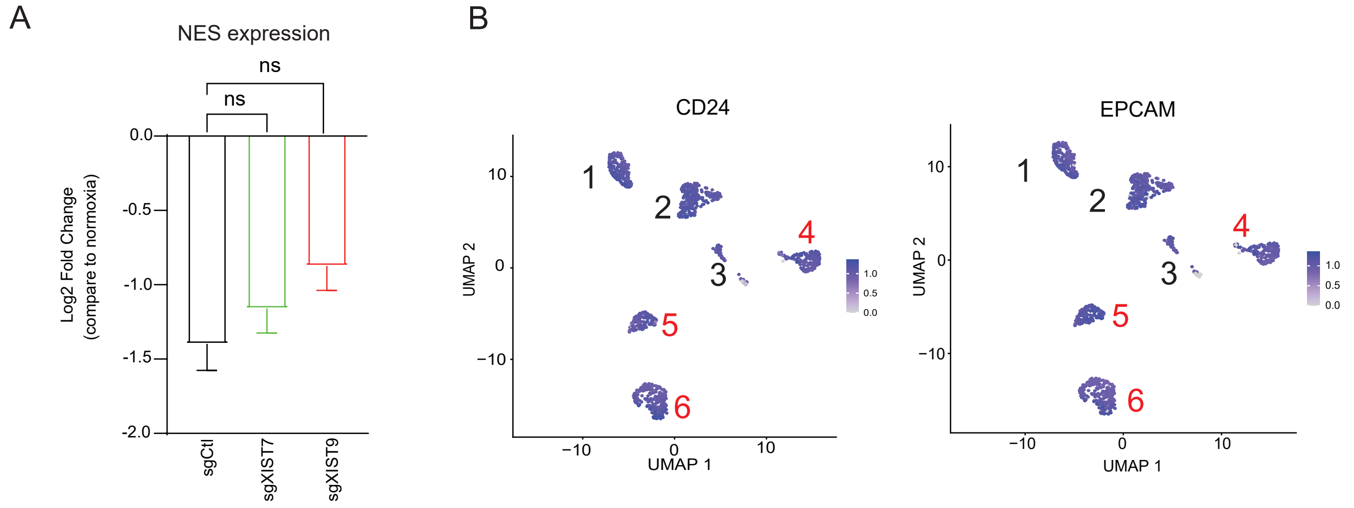

<u>Supplemental Figure 6:</u> (A) Log2 fold change expression of NES after 24h of hypoxia in OVCAR3-KRAB with sgCtl, sgXIST7, and sgXIST9 versus sgCtl, sgXIST7 and sgXIST9 respectively in normoxia condition. **(B)** UMAP maps of CD24 (left) and EPCAM (right) in tumor cells in ovarian cancer patients.
